# Fractional-order differential model for knee implant recovery in smart health infrastructures

**DOI:** 10.1038/s41598-026-48965-7

**Published:** 2026-04-17

**Authors:** Titus Ifeanyi Chinebu, Kennedy Chinedu Okafor, Cajetan Uwatoronye Nwadinigwe, Juliet Onyinye Nwigwe, Diovu Remigius Chidiebere, Okafor Ijeoma Peace, Omowunmi Mary Longe, Kelvin Anoh

**Affiliations:** 1 Federal University of Allied-Health Sciences, Enugu, Nigeria; 2 Imperial College London, South Kensington Campus, London, SW7 2AZ UK; 3 Department of Engineering, Manchester Metropolitan University, Manchester, M1 5GD UK; 4 Department of Electrical and Electronic Engineering Science, University of Johannesburg, Johannesburg, 2006 South Africa; 5 Center for Future Technologies, University of Chichester, Bognor Regis, PO21 1HR UK; 6 Department of Public Health, Cardiff Metropolitan University, Llandaff Campus, Western Avenue, Cardiff, CF5 2YB UK

**Keywords:** Artificial Intelligence Agents, Computational Model, Fractional-order delay model, Inflammation dynamics, IoT-enabled rehabilitation, Patient-centred monitoring, Post-surgical recovery, Engineering, Health care, Medical research

## Abstract

Recovery from knee replacement surgery in conventional orthopaedic healthcare systems can be delayed due to high costs, pain, limited monitoring, and insufficient follow-up, which may hinder early detection of poor healing and inflammation. This study presents a smart orthopaedic healthcare model for knee replacement recovery, where integrated sensors support continuous monitoring and improve the rehabilitation process, thereby reducing delays associated with traditional care systems. A mathematical modelling approach based on compartmental modelling and fractional-order dynamics is used to represent delayed healing responses following total knee replacement surgery. The analysis shows that prolonged delays in monitoring and treatment can lead to unstable recovery patterns, resulting in fluctuations in knee function and inflammation levels. To improve recovery outcomes, the study demonstrates that intelligent sensing devices providing real-time feedback effectively reduce inflammation and enhance joint performance. Results show that when delays are minimised, near-complete restoration of knee function is achieved (≥ 98%), accompanied by optimal inflammation suppression and functional recovery. However, in the presence of delays, recovery remains substantially improved, particularly in terms of inflammation control (≈ 92%), although overall functional gains and recovery efficiency are comparatively reduced. Overall, this work highlights the importance of early inflammation management and feedback-assisted rehabilitation in maintaining knee stability and accelerating recovery. The proposed model provides a theoretical foundation for developing advanced rehabilitation strategies and intelligent device-assisted therapies in smart orthopaedic healthcare systems.

**Supplementary Information:**

The online version contains supplementary material available at 10.1038/s41598-026-48965-7.

## Introduction

Knee replacement is a widely performed procedure worldwide, involving the implantation of artificial components to restore joint function and mobility. Total knee replacement (TKR) is considered the most effective intervention for alleviating pain and improving function in patients with advanced knee osteoarthritis. Surgical intervention is typically indicated when joint degeneration results in persistent pain and significant impairment in daily activities. Demographic trends, including ageing populations, increasing life expectancy, and rising body mass index (BMI), have contributed to a growing demand for knee replacement procedures^1^. Advances in implant design, surgical techniques, and perioperative management continue to enhance clinical outcomes and long-term prosthesis longevity.

Two main approaches exist: TKR, which replaces the entire joint, and partial knee replacement (PKR), which resurfaces only the affected compartment and usually allows faster recovery. A TKR procedure replaces the damaged articular surfaces with prosthetic components, typically including a femoral component, a tibial component, and sometimes a patellar component. Following surgery, recovery proceeds through staged pain management, inflammation control, and gradual restoration of joint mechanics. In the early phase, efforts focus on reducing pain and swelling while initiating gentle mobility exercises^1^. As inflammation subsides, rehabilitation advances to weight-bearing and strengthening activities, supporting progressive functional improvement. Most patients achieve satisfactory recovery within 3–6 months, with full functional restoration potentially extending up to 12 months. Total knee arthroplasty (TKA) remains the standard of care for end-stage osteoarthritis, providing sustained pain relief and improved mobility^2,3^.

Despite general success, complications such as delayed inflammation and mechanical issues can prolong recovery. Recovery is influenced by immune responses, tissue regeneration, and biomechanics, with delayed inflammation being a critical factor^4^. Immune-mediated damage at time $\:t-\tau\:$can persist and impair healing at time $\:t$. Early post-op cytokines like TNF-α, IL-1β, and IL-6 decline quickly, but fibrosis and tissue damage may last weeks. Delayed inflammation, often triggered by wear particles, can lead to aseptic loosening, stiffness, swelling, and pain^1,5^. Biomarkers such as CRP, ESR, IL-6, and TNF-α are used to identify inflammation and guide interventions, including NSAIDs, corticosteroids, IL-6 inhibitors, synovial fluid analysis, and PET-MRI. Mechanical recovery depends on load-bearing, alignment, and muscle strength; malalignment, fibrosis, or persistent inflammation can delay functional restoration. Implant design also affects outcomes, with posterior-stabilised implants improving flexion but stressing posterior structures, while mobile-bearing implants enhance kinematics at a higher dislocation risk. Patient-specific instrumentation (PSI) and robotic-assisted surgery improve alignment and early recovery.

Wearable sensors are increasingly used to monitor joint angles and recovery trajectories, enabling remote patient monitoring and timely interventions^6^. Smart implants with embedded sensors are under development to provide continuous data on pressure, inflammation, and implant performance. Patient acceptance remains variable due to concerns about data privacy and invasiveness, highlighting the need for education and ongoing research.

Author in^7^, illustrates the Fun-Knee™ system, a low-cost, portable solution for monitoring knee angle in TKR rehabilitation. It integrates JY901 altitude modules with an MPU-6050 motion sensor and CC2541 Bluetooth transmitter within a wearable sleeve. High-precision measurement is achieved via a Kalman filter and Digital Motion Processor, accurate to 0.05 degrees. The rechargeable lithium battery supports four hours of use with a two-hour recharge time, making it suitable for daily clinical or home-based physiotherapy^7^.

Recent advances in orthopaedic surgery demonstrate a growing convergence of smart technologies, sensorised systems, and AI-based decision support systems aimed at enhancing surgical precision and postoperative recovery. Frassati et al.^8^. proposed a near-field resonant inductive coupling system to wirelessly power smart implants, reducing the dependency on battery replacement. Eskandari and Lee^9^ developed a decision support platform that integrates clinical outcome measurements to optimise joint replacement aftercare. Mixed reality (MR) and AI integration, as presented by Moglia et al.^10^, facilitates multimodal visualisation during knee osteotomy planning, while Hu et al.^11^ extended this direction with an AR-based markerless navigation framework that minimises invasiveness and improves accuracy. In parallel, Kosmas et al.^12^ designed a sensorised 3D-printed gap gauge to improve prosthesis alignment and long-term durability, and Zhang et al.^13^ introduced SLAM-TKA, a localisation and mapping approach for accurate pin and X-ray positioning during total knee arthroplasty. Beyond knee interventions, Deleu et al.^14^. highlighted biomechanical workload redistribution after ankle replacement, emphasising the value of quantitative biomechanical monitoring in joint replacement surgery. Collectively, these studies highlight a paradigm shift towards precision, personalisation, and continuous monitoring in orthopaedic interventions.

Computational modelling consolidates these innovations by linking mechanical and biological perspectives. Multimodal rehabilitation, including pain management, neuromuscular stimulation, and tailored therapies, remains sensitive to delayed inflammatory responses and mechanical instability. Fractional-order models capture memory effects in immune responses, offering a framework for modelling delayed inflammation in orthopaedic recovery^15^. For example, elevated IL-6 after TKA correlates with poorer long-term outcomes^16^, and chronic low-grade inflammation slows mechanical recovery^17^. Delay differential equations (DDEs) provide a foundation for modelling systems whose present state depends on past dynamics^18,19^. Their fractional extension, fractional delay differential equations (FDDEs), generalises to non-integer orders, capturing small-delay effects and long-term memory, with established results in existence, stability, and controllability^20–24^.

Fractional-order differential equations (FDEs) are particularly relevant in bioengineering due to viscoelastic tissue behaviour and long-range physiological dependencies^25–27^ and have been applied in tumour–immune interactions^28,30^ and broader bioengineering contexts^31–38^. Advanced numerical methods, including Mittag–Leffler functions, Laplace transforms, and fractional collocation schemes, facilitate simulation of complex, time-dependent physiological processes^39–46^. Despite these advances, existing TKR recovery models primarily rely on integer-order dynamics, neglecting critical features such as tissue memory, delayed immune and neuromuscular responses, and patient-specific variability. They often fail to account for hereditary properties of ligaments, tendons, and periarticular soft tissues, or time-lagged effects from inflammation and immune signalling, which strongly influence post-surgical outcomes. These insights motivate the integration of sensor-based monitoring, predictive modelling, and adaptive feedback in smart rehabilitation systems.

To address these gaps, this work introduces a fractional-order delay computational model (FODCM) for knee implant recovery. Its novelty lies in combining fractional calculus with time-delay components to capture: (i) biomechanical memory effects from prior mechanical stress and inflammation, (ii) physiological delays including tissue remodelling, immune adaptation, and neuromuscular feedback, and (iii) nonlinear, patient-specific dynamics to simulate individualised rehabilitation and device-assisted interventions. Unlike traditional models, the FODCM framework enables real-time monitoring and prediction of recovery dynamics, including oscillatory instabilities under conditions of high delay or elevated inflammation. It also supports quantitative evaluation of sensing-assisted rehabilitation strategies. By incorporating memory effects, adaptive recovery behaviour, and delayed physiological responses, the model addresses key limitations in existing orthopaedic personalised post-surgical care for knee implant patients. Furthermore, artificial intelligence (AI)-driven agents can be integrated into this framework to support automated decision-making, real-time feedback, and adaptive rehabilitation planning, thereby enhancing clinical efficiency and patient outcomes.

The paper is structured as follows: Sect.  2 presents the system model and problem formulation for knee implant recovery. Section  3 investigates the local stability of equilibrium points, including inflammation-free and persistent states. Section  4 analyses the existence of critical delays and Hopf bifurcation phenomena. Section  5 provides numerical simulations and model validation, illustrating the dynamics of post-surgical recovery, inflammation, and implant mechanical function under various biological and environmental conditions. Finally, Sect.  6 concludes the study and discusses implications for rehabilitation and future research.

## System model and problem formulation

**Fig. 1:**
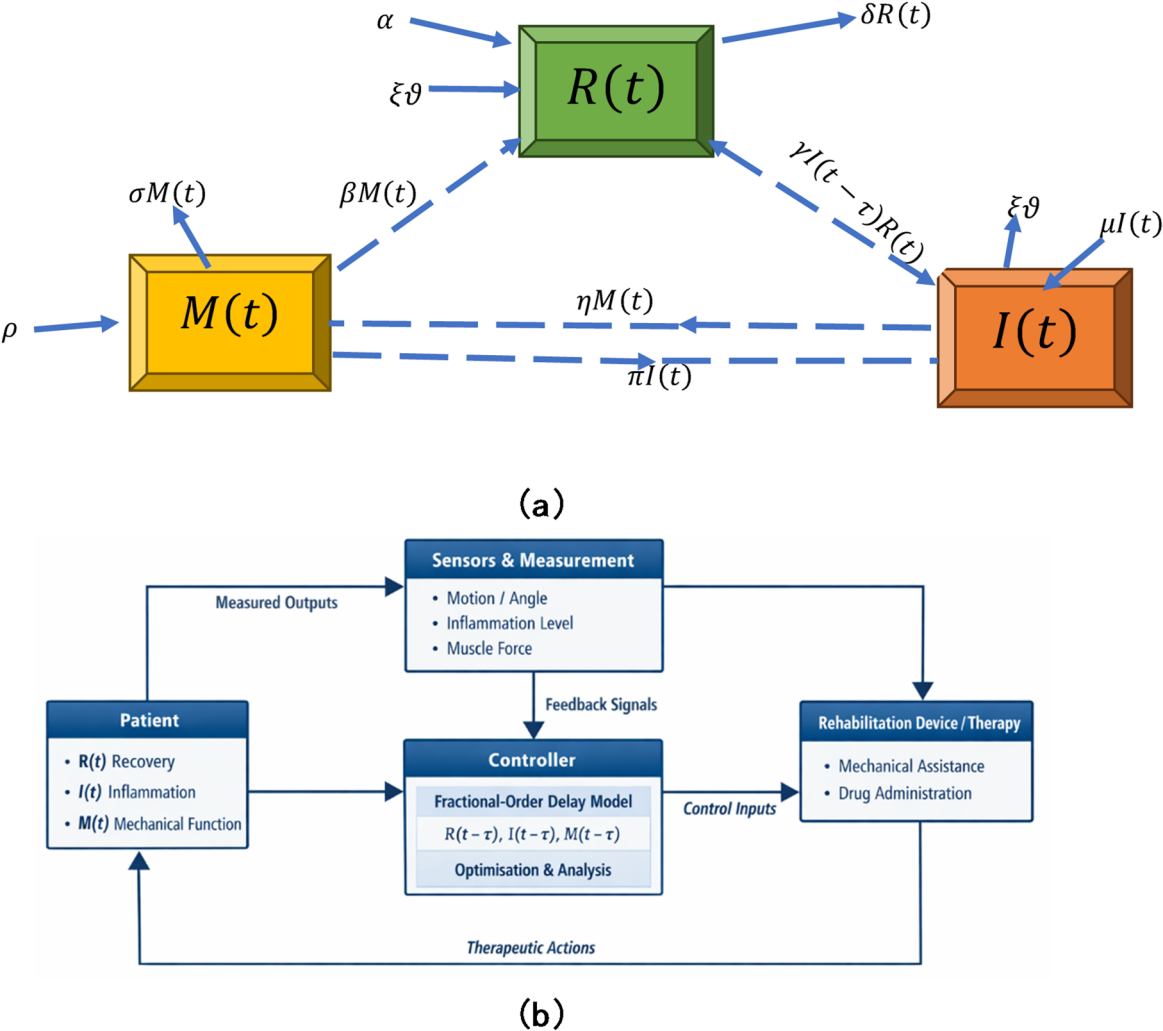
(**a**) Schematic diagram of the Knee implant surgery delay mode. (**b**) Closed-loop diagram of the rehabilitation device, the Knee implant surgery delay mode.

This section introduces the mathematical characterization of recovery dynamics in patients following total knee replacement in Fig. 1a and b. The formulation integrates mechanical, inflammatory, and feedback-delay processes into a unified fractional-order delay computational model (FODCM). The objective is to capture the interplay between prosthetic joint mechanics, immune response, and time-dependent recovery effects, thereby enabling stability analysis and predictive evaluation of rehabilitation trajectories.

### Nomenclature

Tables 1 and 2 define the variables and parameters used in the knee implant model, while Table 3 summarises model features alongside their clinical and biomechanical justifications.

**Table 1:** System model variables.

Variable	Description
*R* $\:\left(t\right)$	Recovery Dynamics through Rehabilitation progress at time *t.* Degree of tissue healing and physiological restoration. Represents the functional recovery level or muscle strength gain of the patient post-knee implant surgery.
$\:I\left(t\right)$	Inflammation Dynamics or immune response level at time *t*. Local/systemic inflammatory response post-surgery. Indicates how much inflammation is present, possibly due to immune response to the implant or surgical procedure.
$\:M\left(t\right)$	Mechanical Function of the Implant, that is, physical joint capability or usability at time *t*. Reflects the concentration or effectiveness of post-operative medication (e.g., anti-inflammatory drugs or antibiotics), or it can also represent mechanical therapy such as physiotherapy.

**Table 2:** Model parameters.

Parameters	Description
$\:\alpha\:$	Baseline recovery rate, that is, initial or innate tissue repair (e.g., cellular healing without therapy).
$\:\beta\:$	Mechanical enhancement of recovery, that is, stronger knee function, improves ability to heal.
$\:\delta\:$	Natural loss or degradation of recovery. Degeneration or poor rehabilitation causes recovery to fade.
$\:\mu\:$	Inflammation self-propagation rate. Immune response sustaining itself (e.g., via cytokines).
$\:\gamma\:$	Inflammatory inhibition factor, meaning that past inflammation suppresses current healing
$\:\eta\:$	Mechanical reduction of inflammation. Better movement drains swelling and boosts circulation.
$\:\rho\:$	External stimulus to mechanical function. Therapy, assistive devices, or prosthetic design
$\:\pi\:$	Inflammatory suppression of mobility. Swelling, pain, stiffness, and reduced joint use.
$\:\sigma\:$	Natural decline of mechanical function, that is, Fatigue, disuse, or biomechanical loss.
$\:\vartheta\:$	Modified parameters that account for the rate at which data captured from knee implant surgery patients are transmitted in real time to healthcare providers.
$\:\xi\:$	The rate at which biosensors of the wearable devices collect data from knee implant surgery patients, that is, real-time monitoring.
$\:\tau\:$	Time delay in physiological response. Captures the delay between treatment or inflammation and its observable impact on rehabilitation.

**Table 3:** Model features and justification.

Feature	Purpose	Biological justification
Delay in inflammation effect on recovery $\:I(t-\tau\:)$	Captures biological lag in immune suppression of healing	Cytokines and immune cells take time to manifest effects
Use of current $\:R\left(t\right)$ in inflammation reduction	Model’s real-time recovery mechanisms fighting residual inflammation	Therapy boosts immune modulation directly
Mechanical function is affected by all components	Combines joint healing, immune condition, and therapy	Reflects clinical rehab outcome trajectories
External therapy input	Allows modelling various rehab regimens	Used in optimal control or scenario analysis

As illustrated in Fig. 1b, the closed-loop knee implant recovery system integrates the patient, sensors, controller, and rehabilitation device into a unified feedback framework. The patient block captures recovery dynamics *R*$\:\left(t\right)$, inflammation *I*$\:\left(t\right)$ and mechanical function *M*$\:\left(t\right)$, which are measured by wearable sensors and devices monitoring motion, joint angles, muscle force, and inflammation levels. These measurements feed into a fractional-order delay controller, which optimises therapy and predicts recovery trajectories. It also accounts for the memory effects and physiological delays. The controller generates control input for mechanical assistance or pharmacological interventions delivered through the rehabilitation device. This, in turn, influences the patient’s recovery, completing the feedback loop. The system enables real-time, personalised rehabilitation, while combining biomechanical, immunological, and delayed response dynamics.

Importantly, the framework is designed to complement existing sensor-based rehabilitation platforms rather than replace them. Home-based wearable technologies, such as the MotionSense wearable system, enable frequent monitoring and timely feedback, while in-clinic motion analysis systems, such as the KneeKG system, provide high-precision assessments during scheduled clinical visits. By modelling feedback delays across these environments, the proposed system enables unified interpretation of recovery data, allowing consistent comparisons between home and clinical monitoring settings. These findings provide insights into how continuous and intermittent monitoring strategies may influence long-term recovery stability and inflammation dynamics.

### Preliminaries

In this section, we provide basic definitions of Louville – Caputo and Caputo – Fabrizio variable order fractional derivatives, which are used in the subsequent sections to characterise Fig. 1b. These preliminaries are essential because variable-order fractional derivatives rigorously capture the system’s memory, delayed responses, and patient-specific dynamics. This ensures accurate modelling, stable closed-loop behaviour, and effective optimisation of personalised rehabilitation protocols.

#### Definition 1

The Louville – Caputo fractional derivative with variable order $\:{\mathcal{q}}_{i}$ is defined as

1 $$\:{{}_{0}{}^{C}\mathcal{D}}_{t}^{\mathcal{q}}\left(f\left(t\right)\right)=\frac{1}{{\Gamma\:}\left(1-\mathcal{q}\right)}\underset{0}{\overset{t}{\int\:}}{\left(t-s\right)}^{-\mathcal{q}}f\left(s\right)ds$$

#### Definition 2

The Caputo – Fabrizio derivative with variable -order If $\:\:{\mathcal{q}}_{i}\:$in Liouville- Caputo sense (CFC)^47^ is defined as follows:

2 $$\:{{}_{0}{}^{CFC}\mathcal{D}}_{t}^{\mathcal{q}}\left(f\left(t\right)\right)=\frac{\left(2-\mathcal{q}\right)\mathcal{W}\left(\mathcal{q}\right)}{2\left(1-\mathcal{q}\right)}\underset{0}{\overset{t}{\int\:}}{f}^{{\prime\:}}\left(s\right)\mathrm{exp}\left[-\mathcal{q}\frac{t-s}{1-\mathcal{q}}\right]ds\:\:\:\:\:0<{\mathcal{q}}_{i}<1$$

In the above expression, $\:\mathcal{W}\left(\mathcal{q}\right)=\frac{2}{2-\mathcal{q}}$ is a normalization of the function that satisfies the condition $\:\mathcal{W}\left(0\right)=\mathcal{W}\left(1\right)=1$ presented by Losada and Nieto^48^.

But If $\:x\in\:{\mathcal{H}}^{1}\left(\mathcal{g},\mathcal{\:}\mathcal{h}\right),\mathcal{\:}\mathcal{h}>\mathcal{g},\mathcal{\:}\mathcal{q}\in\:\left[0,\:1\right],\:$then the new fractional derivative of arbitrary order can be defined as

3 $$\:{{}_{0}{}^{CFC}\mathcal{D}}_{t}^{\mathcal{q}}\left(f\left(t\right)\right)=\frac{\left(2-\mathcal{q}\right)\mathcal{W}\left(\mathcal{q}\right)}{2\left(1-\mathcal{q}\right)}\underset{\mathcal{g}}{\overset{t}{\int\:}}\left(f\left(t\right)-f\left(s\right)\right)\mathrm{exp}\left[-\mathcal{q}\frac{t-s}{1-\mathcal{q}}\right]ds$$

#### *Remark 1*

If $\:\varphi\:=\frac{1-\mathcal{q}}{\mathcal{q}}\in\:\left[0,\infty\:\right),\:\mathcal{q}=\frac{1}{1+\varphi\:}\in\:\left[0,\:1\right],$ then Eq. (3) presumes the form

4 $$\:{\mathcal{D}}_{t}^{{\mathcal{q}}_{i}\left(t\right)}\left(f\left(t\right)\right)=\frac{N\left(\zeta\:\right)}{\zeta\:}\underset{\mathcal{g}}{\overset{t}{\int\:}}{f}^{{\prime\:}}\left(s\right)\mathrm{exp}\left[-\frac{t-s}{\zeta\:}\right]ds,\:\:\:N\left(0\right)=N\left(\infty\:\right)=1$$

Moreover,

5 $$\mathop {{\mathrm{lim}}}\limits_{{\phi \to 0}} \frac{1}{\phi }exp\left[ { - \frac{{t - s}}{\phi }} \right] = \xi \left( {s - t} \right)$$

The corresponding fractional integral resulted in being essential^48^.

### System model equations

This subsection presents the system model equations, using variable-order fractional derivatives and a delay term or physiological response time $\:\tau\:$ to capture the coupled dynamics of recovery, inflammation, and implant mechanical function during personalised rehabilitation.

The model variables are formulated using a first-order fractional dynamic framework, which captures the memory-dependent and cumulative nature of physiological rehabilitation, rather than oscillatory or inertial behaviour. The recovery state $\:R\left(t\right)$ in Equ (6a), represents functional rehabilitation progress and corresponds to PROMs, pain score improvement, and cumulative activity levels from wearable sensors. The inflammatory state $\:I\left(t\right)$ in Equ (6b) reflects immune responses and maps to swelling, local temperature, inflammatory biomarkers, or sensor-based proxies such as limb circumference and thermal imaging. The mechanical function state $\:M\left(t\right)$ in Equ (6c) describes biomechanical recovery. It maps to the range of motion, gait kinematics, cadence, step count, and force/pressure distribution measured via wearables or instrumented walkways.

- Recovery Dynamics through *Rehabilitation progress* at time *t*.
  6 $$\:{{}_{0}{}^{CFC}\mathcal{D}}_{t}^{\mathcal{q}}R\left(t\right)=\alpha\:+\xi\:\vartheta\:+\beta\:M\left(t\right)-\gamma\:I\left(t-\tau\:\right)R\left(t\right)-\delta\:R\left(t\right)$$
- Inflammation Dynamics *or immune response level* at time t.
  7 $$\:{{}_{0}{}^{CFC}\mathcal{D}}_{t}^{\mathcal{q}}I\left(t\right)=\mu\:I\left(t\right)-\gamma\:I\left(t-\tau\:\right)R\left(t\right)-\eta\:M\left(t\right)-\xi\:\vartheta\:$$
- Mechanical Function of the Implant *using Medication or therapy level* at time t.
  8 $$\:{{}_{0}{}^{CFC}\mathcal{D}}_{t}^{\mathcal{q}}M\left(t\right)=\rho\:-\pi\:I\left(t\right)-\sigma\:M\left(t\right)$$

The monitoring is described in continuous time, representing a high-frequency sensing system that allows timely feedback and intervention. In practice, measurements may happen at discrete intervals, such as daily wearable checks or periodic clinic visits. The framework accounts for this by treating the sensing effectiveness parameter (e.g., ξϑ) as a summary of measurement frequency, data quality, and feedback responsiveness. Checking the patient less often can slow recovery and let inflammation persist, while more frequent monitoring supports faster improvement and stabilises the patient’s condition. Thus, the continuous-time formulation provides a flexible approximation applicable across practical monitoring schedules, while the sensing effectiveness parameter summarises the combined influence of frequency, quality, and responsiveness on recovery dynamics.

### Model interpretation

A delay differential equation (DDE) is a type of differential equation where the derivative of a function at a certain time depends on its values at previous times. This delay accounts for real-world phenomena where responses to changes do not happen instantaneously but rather after some time lag. For the DDE model of knee implant surgery recovery, we have the recap of the knee implant model (with Delay) given in Equ 7.

9 $$\:\left.\begin{array}{c}{{}_{0}{}^{CFC}\mathcal{D}}_{t}^{\mathcal{q}}R\left(t\right)=\alpha\:+\xi\:\vartheta\:+\beta\:M\left(t\right)-\gamma\:I\left(t-\tau\:\right)R\left(t\right)-\delta\:R\left(t\right),\\\:{{}_{0}{}^{CFC}\mathcal{D}}_{t}^{\mathcal{q}}I\left(t\right)=\mu\:I\left(t\right)-\gamma\:I\left(t-\tau\:\right)R\left(t\right)-\eta\:M\left(t\right)-\xi\:\vartheta\:,\\\:{{}_{0}{}^{CFC}\mathcal{D}}_{t}^{\mathcal{q}}M\left(t\right)=\rho\:-\pi\:I\left(t\right)-\sigma\:M\left(t\right).\end{array}\right\}$$

With the initial values$\:\:R\left(0\right)={R}^{*},\:I\left(0\right)={I}^{*},\:M\left(0\right)={M}^{*};\:{\Omega\:}\in\:\left[-\tau\:,\:0\right]$.

Where the parameter $\:\tau\:\:$denotes the physiological and rehabilitation response delay, measured in days. It represents the time lag between inflammatory activity and its observable impact on functional recovery following knee implant intervention. This delay does not correspond to a monitoring or observation window; rather, it captures the latent biological and mechanical processes—such as inflammation resolution, tissue remodeling, neuromuscular adaptation, and rehabilitation response—that require time to influence recovery outcomes. The wearable-related parameter $\:\xi\:\vartheta\:$ characterises the effectiveness of real-time sensing and feedback in supporting recovery and is independent of the delay duration. The delayed coupling within the model reflects the interconnected nature of post-surgical recovery dynamics. In particular, past inflammation $\:I(t-\tau\:)$ negatively affects current recovery $\:R\left(t\right)$, indicating that residual swelling, pain, or oxidative stress can slow healing and cause temporary setbacks. Conversely, improved recovery contributes to the gradual reduction of inflammation, as ongoing healing and restored mechanical function promote immune deactivation. These bidirectional negative interactions highlight the non-instantaneous feedback between inflammation and recovery. Clinically, the delay $\:\tau\:$ corresponds to realistic post-operative and rehabilitation time scales, typically ranging from several days to weeks, depending on patient-specific factors and treatment intensity. To assess how such delays influence system stability and recovery outcomes after knee implant surgery, the characteristic equation is derived from the Jacobian matrix of the linearised Equ (7) evaluated at the equilibrium point.

### Equilibrium points (inflammation-free and persistent)

In this subsection, we examine the existence of equilibrium points. Using model (7), we perform a stability analysis to identify the inflammation-free and persistent equilibrium states. To find these points, each equation in (7) is set to zero in order to establish these equilibrium points.

10 $$\:\left.\begin{array}{c}0=\alpha\:+\xi\:\vartheta\:+\beta\:M\left(t\right)-\gamma\:I\left(t-\tau\:\right)R\left(t\right)-\delta\:R\left(t\right),\\\:0=\mu\:I\left(t\right)-\gamma\:I\left(t-\tau\:\right)R\left(t\right)-\eta\:M\left(t\right)-\xi\:\vartheta\:,\\\:0=\rho\:-\pi\:I\left(t\right)-\sigma\:M\left(t\right).\end{array}\right\}$$

Also, at equilibrium, all state variables are constant, so:

11 $$\:R\left(t\right)={R}^{*},\:I\left(t\right)={I}^{*},\:M\left(t\right)={M}^{*}$$

And delays don’t matter anymore because:

12 $$\:\:I\left(t-\tau\:\right)={I}^{*}$$

Now, the equilibrium points of $\:R\left(t\right),\:I\left(t\right),\:M\left(t\right)$ is determined. The equilibrium where the rate of inflammation is zero is the so-called inflammation-free equilibrium (IFE). When there isn’t inflammation due to knee implant surgery, the conditions that define the inflammation-free equilibrium are met, which means $\:I\left(t\right)\:=\:0$. Using (8), we obtain $\:S\left(t\right)\:and\:M\left(t\right)$. Therefore, the inflammation-free equilibrium points for the knee implant surgery delay differential equation model are: $\:{E}^{*}=\left({R}^{*},\:{I}^{*},\:{M}^{*}\right)=\left(\frac{\sigma\:\left(\alpha\:+\xi\:\vartheta\:\right)+\beta\:\rho\:}{\delta\:\sigma\:},\:0,\frac{\rho\:}{\sigma\:}\right)$.

Solving the system of Eq. (10), we have from the third equation that:

13 $$\:0 = \rho \: - \sigma \:M^{*} \Rightarrow M^{*} = \frac{{\rho \:}}{{\sigma \:}}$$

From the first equation of system (8), we have

14 $$\:\alpha \: + \xi \:\vartheta \: + \beta \:M^{*} - \delta \:R^{*} = 0 \Rightarrow R^{*} = \frac{{\alpha \: + \xi \:\vartheta \: + \beta \:M^{*} }}{{\delta \:}}$$

Substitute (11) into system (12), we have:

15 $$\:{R}^{*}=\frac{\sigma\:\left(\alpha\:+\xi\:\vartheta\:\right)+\beta\:\rho\:}{\delta\:\sigma\:}$$

Then we the IFE summary as

16 $$\:\left({R}^{*},\:{M}^{*},\:{I}^{*}\right)=\left(\frac{\sigma\:\left(\alpha\:+\xi\:\vartheta\:\right)+\beta\:\rho\:}{\delta\:\sigma\:},\:0,\frac{\rho\:}{\sigma\:}\right)$$

By taking into account the scenario in which $\:I\left(t\right)$ is positive, we can identify the inflammation persistent equilibria of the delay model. Inflammation persistent equilibrium (IPE) means a steady state level of inflammation that does not resolve (i.e., the inflammatory response becomes chronic instead of healing and returning to baseline). Here, pro inflammatory stimuli (like wear particles, bacterial biofilm, or immune triggers) continually activate the immune system. The anti-inflammatory mechanisms fail to suppress or resolve the inflammation. This creates a stable, non-zero level of inflammatory mediators and immune activity. Solving for $\:R\left(t\right)\:I\left(t\right)\:and\:M(t$) in Equ (8), the inflammation persistent equilibrium points for the delay differential equation model were determined. From third Equ (8) we have

17 $$\:{M}^{**}=\frac{\rho\:-\pi\:{I}^{**}}{\sigma\:}$$

From the first equation of system (8) we obtain

18 $$\:{R}^{**}=\frac{\sigma\:\left(\alpha\:+\xi\:\vartheta\:\right)+\beta\:\left(\rho\:-\pi\:{I}^{**}\right)}{\sigma\:\left(\gamma\:{I}^{**}+\delta\:\right)}$$

The second equation of system (2) gives

19 $$\begin{aligned} & \mu I^{{**}} - \gamma I^{{**}} R^{*} = \xi \vartheta + \eta M^{{**}} \Rightarrow I^{{**}} = \frac{{\xi \vartheta + \eta \left( {\frac{{\rho - \pi I^{{**}} }}{\sigma }} \right)}}{{\left( {\mu - \gamma \left( {\frac{{\sigma \left( {\alpha + \xi \vartheta } \right) + \beta \left( {\rho - \pi I^{*} } \right)}}{{\sigma \left( {\gamma I^{*} + \delta } \right)}}} \right)} \right)}} \\ & I^{{**}} = \frac{{\frac{{\sigma \:\xi \:\vartheta \: + \eta \:\left( {\rho \: - \pi \:I^{{**}} } \right)}}{{\sigma \:}}}}{{\left( {\frac{{\mu \:\sigma \:\left( {\gamma \:I^{{**}} + \delta \:} \right) - \gamma \:\left( {\sigma \:\left( {\alpha \: + \xi \:\vartheta \:} \right) + \beta \:\left( {\rho \: - \pi \:I^{{**}} } \right)} \right)}}{{\sigma \:\left( {\gamma \:I^{{**}} + \delta \:} \right)}}} \right)}} \\ & \quad \Rightarrow I^{{**}} = \frac{{\left( {\sigma \:\xi \:\vartheta \: + \eta \:\left( {\rho \: - \pi \:I^{{**}} } \right)} \right)\left( {\gamma \:I^{{**}} + \delta \:} \right)}}{{\left( {\mu \:\sigma \:\left( {\gamma \:I^{{**}} + \delta \:} \right) - \gamma \:\left( {\sigma \:\left( {\alpha \: + \xi \:\vartheta \:} \right) + \beta \:\left( {\rho \: - \pi \:I^{{**}} } \right)} \right)} \right)}} \\ & \quad \Rightarrow \gamma \:\left[ {\mu \:\sigma \: + \beta \:\pi \: + \eta \:\pi \:} \right]I^{{**2}} + \left[ {\mu \:\sigma \:\delta \: + \eta \:\delta \:\pi \: - \gamma \:\alpha \:\sigma \: - \gamma \:\beta \:\rho \: - \eta \:\gamma \:\rho \: - 2\gamma \:\sigma \:\xi \:\vartheta \:} \right]I^{{**}} \\ & \quad - \delta \:\rho \:\eta \: - \sigma \:\delta \:\xi \:\vartheta \: = 0 \\ \end{aligned}$$

Rewriting Eq. (19) gives

$$\:{\mathcal{a}}_{2}{{I}^{**}}^{2}+{\mathcal{a}}_{1}{I}^{**}+{\mathcal{a}}_{0}=0$$

Where $$a_{2} = \gamma \left[ {\mu \sigma + \beta \pi + \eta \pi } \right]$$, $$a_{1} = \left[ {\mu \sigma \delta + \eta \delta \pi - \gamma \alpha \sigma - \gamma \beta \rho - \eta \gamma \rho - 2\gamma \sigma \xi \vartheta } \right]$$ and $$a_{0} = - \delta \rho \eta - \sigma \delta \xi \vartheta$$

Therefore, letting $$\:{I}^{**}$$ be the knee implant surgery inflammation persistent equilibrium of the delay model, we have

20 $$\:{E}^{**}=\left({R}^{**},\:{I}^{**},\:{M}^{**}\right)$$

The characteristic equation of the Jacobian matrix of the linearized model system (7) at the equilibrium is given as

21 $$\:\left[\begin{array}{ccc}-\delta\:-\gamma\:\widehat{I}-\lambda\:&\:-{\gamma\:\widehat{R}e}^{-\lambda\:\tau\:}&\:\beta\:\\\:-\gamma\:\widehat{I}&\:\mu\:-{\gamma\:\widehat{R}e}^{-\lambda\:\tau\:}-\lambda\:&\:-\eta\:\\\:0&\:-\pi\:&\:-\sigma\:-\lambda\:\end{array}\right]=0$$

Since the steady state about which we have linearized is stable in the absence of delay, that is when $\:\tau\:=0,$which simply implies that there is no delay and that all the roots of the polynomial have non positive real parts. We further consider the situation where $\:\tau\:>0$ and therefore substitute the steady state $\:\left(\frac{\sigma\:\left(\alpha\:+\xi\:\vartheta\:\right)+\beta\:\rho\:}{\delta\:\sigma\:},\:0,\frac{\rho\:}{\sigma\:}\right)$ into system (19), after which we obtain the characteristic equation as;

22 $$\:\left[\begin{array}{ccc}-\delta\:-\lambda\:&\:-{\gamma\:\left(\frac{\sigma\:\left(\alpha\:+\xi\:\vartheta\:\right)+\beta\:\rho\:}{\delta\:\sigma\:}\right)e}^{-\lambda\:\tau\:}&\:\beta\:\\\:0&\:\mu\:-{\gamma\:\left(\frac{\sigma\:\left(\alpha\:+\xi\:\vartheta\:\right)+\beta\:\rho\:}{\delta\:\sigma\:}\right)e}^{-\lambda\:\tau\:}-\lambda\:&\:-\eta\:\\\:0&\:-\pi\:&\:-\sigma\:-\lambda\:\end{array}\right]=0$$

23 $$\begin{aligned} & \lambda \:^{3} + \left( {\delta \: + \sigma \: + \gamma \:\left( {\frac{{\sigma \:\left( {\alpha \: + \xi \:\vartheta \:} \right) + \beta \:\rho \:}}{{\delta \:\sigma \:}}} \right)e^{{ - \lambda \:\tau \:}} - \mu \:} \right)\lambda \:^{2} + \left( {\delta \:\sigma \: + \delta \:\gamma \:\left( {\frac{{\sigma \:\left( {\alpha \: + \xi \:\vartheta \:} \right) + \beta \:\rho \:}}{{\delta \:\sigma \:}}} \right)e^{{ - \lambda \:\tau \:}} } \right. \\ & \left. { + \sigma \:\gamma \:\left( {\frac{{\sigma \:\left( {\alpha \: + \xi \:\vartheta \:} \right) + \beta \:\rho \:}}{{\delta \:\sigma \:}}} \right)e^{{ - \lambda \:\tau \:}} - \eta \:\pi \: - \delta \:\mu \: - \mu \:\sigma \:} \right)\lambda \: + \delta \:\sigma \:\gamma \:\left( {\frac{{\sigma \:\left( {\alpha \: + \xi \:\vartheta \:} \right) + \beta \:\rho \:}}{{\delta \:\sigma \:}}} \right)e^{{ - \lambda \:\tau \:}} \\ & - \pi \:\delta \:\eta \: - \delta \:\mu \:\sigma \: = 0 \\ \end{aligned}$$

System (21) can be reduced to

24 $$\:{a}_{i}\left(\lambda\:\right)+{h}_{i}\left(\lambda\:\right){e}^{-\lambda\:\tau\:}=0$$

25 $$\:{\lambda\:}^{3}+{a}_{1}{\lambda\:}^{2}+{a}_{2}\lambda\:+{a}_{3}+\left({h}_{1}{\lambda\:}^{2}+{h}_{2}\lambda\:+{h}_{3}\right){e}^{-\lambda\:\tau\:}=0$$

### Critical delay model and Hopf bifurcation existence

In this section, we investigate the existence of a critical time delay $\:{\tau\:}_{c}>0\:$for which the real part of an eigenvalue becomes positive when $\:\tau\:>{\tau\:}_{c}$. This threshold delay corresponds to the moment when Equ (23) shifts from having eigenvalues with non-positive real parts to admitting eigenvalues with non-negative real parts. Equivalently,$\:{\tau\:}_{c}$is defined as the value of the delay parameter at which $\:\mathrm{R}\mathrm{e}\left(\lambda\:\right)=0,$ signaling a change in system behavior from stability to instability. To examine whether such a delay value exists, Equ (23) is reformulated into an equivalent polynomial representation. This allows us to analyze the nature of its roots and assess the possibility of bifurcation induced by the delay. Our analysis begins by considering the presence of purely imaginary eigenvalues. Let $\:\lambda\:\left(\tau\:\right)=\zeta\:\left(\tau\:\right)+i\varphi\:\left(\tau\:\right),$ where $\:\zeta\:\left(\tau\:\right)$and $\:\varphi\:\left(\tau\:\right)$are real-valued functions of $\:\tau\:$. At $\:\tau\:=0$, we have $\:\zeta\:\left(0\right)<0$. By the continuity of $\:\zeta\:\left(\tau\:\right)$, there exists an interval $\:0\le\:\tau\:\le\:{\tau\:}_{c}$for some $\:{\tau\:}_{c}>0$ such that $\:\zeta\:\left(\tau\:\right)<0$ throughout this interval. Consequently, the equilibrium point remains stable for all admissible delay values within this range. Substituting $\:\lambda\:\left(\tau\:\right)$ into Equ (23) then yields the following expression (see^50^:

$$\:{\left(\zeta\:+i\phi\:\right)}^{3}+{a}_{1}{\left(\zeta\:+i\phi\:\right)}^{2}+{a}_{2}\left(\zeta\:+i\phi\:\right)+{a}_{3}+\left({h}_{1}{\left(\zeta\:+i\phi\:\right)}^{2}+{h}_{2}\left(\zeta\:+i\phi\:\right)+{h}_{3}\right){e}^{-\tau\:\left(\zeta\:+i\phi\:\right)}=0$$

$$\:{\zeta\:}^{3}+3{\zeta\:}^{2}i\phi\:-3\zeta\:{\phi\:}^{2}-{i\phi\:}^{3}+{a}_{1}{\zeta\:}^{2}+2{a}_{1}\zeta\:i\phi\:-{{a}_{1}\phi\:}^{2}+\:{a}_{2}\zeta\:+{a}_{2}i\phi\:+{a}_{3}$$

$$\:+\left[{{h}_{1}\zeta\:}^{2}+2{h}_{1}\zeta\:i\phi\:-{h}_{1}{\phi\:}^{2}+{h}_{2}\zeta\:+{h}_{2}i\phi\:+{h}_{3}\right]{e}^{-\tau\:\left(\zeta\:+i\phi\:\right)}=0.$$

We probably write the exponential in terms of trigonometric function, that is

But

$$\begin{aligned} & e^{{ - \tau \:\left( {\zeta \: + i\phi \:} \right)}} = e^{{ - \tau \:\zeta \:}} \cdot e^{{ - \tau \:i\phi \:}} , \\ & e^{{ - \tau i\phi }} = {\mathrm{cos}}\tau \phi - isin\tau \phi , \\ & \Rightarrow e^{{ - \tau \zeta }} \cdot e^{{ - \tau i\phi }} = e^{{ - \tau \zeta }} \left( {{\mathrm{cos}}\tau \phi - isin\tau \phi } \right). \\ \end{aligned}$$

Then we break the polynomial up into its real and imaginary parts, and write the exponential in terms of the trigonometric functions to get

26 $$\begin{aligned} & a_{3} + a_{2} \zeta \: + a_{1} \zeta \: + \zeta \: - a_{1} \zeta \:^{2} - 3\zeta \:\phi \:^{2} + i\left( {a_{2} \phi \: + 2a_{1} \zeta \:\phi \: + 3\zeta \:^{2} \phi \: - \phi \:^{3} } \right) \\ & \quad + e^{{ - \tau \:\zeta \:}} \left( {{\mathrm{cos}}\tau \:\phi \: - isin\:\tau \:\phi \:} \right)\left[ {h_{1} \zeta \:^{2} + 2h_{1} \zeta \:i\phi \: - h_{1} \phi \:^{2} + h_{2} \zeta \: + h_{2} i\phi \: + h_{3} } \right] = 0 \\ & \Rightarrow a_{3} + a_{2} \zeta \: + a_{1} \zeta \:^{2} + \zeta \:^{3} - a_{1} \phi \:^{2} - 3\zeta \:\phi \:^{2} + i\left( {a_{2} \phi \: + 2a_{1} \phi \: + 3\zeta \:^{2} \phi \: - \phi \:^{3} } \right) \\ & \quad + e^{{ - \tau \:\zeta \:}} \left[ {{\mathrm{cos}}\tau \:\phi \:\left( {h_{1} \zeta \:^{2} + 2h_{1} \zeta \:i\phi \: - h_{1} \phi \:^{2} + h_{2} \zeta \: + h_{2} i\phi \: + h_{3} } \right)} \right] \\ & \quad - e^{{ - \tau \:\zeta \:}} \left[ {isin\:\tau \:\phi \:\left( {h_{1} \zeta \:^{2} + 2h_{1} \zeta \:i\phi \: - h_{1} \phi \:^{2} + h_{2} \zeta \: + h_{2} i\phi \: + h_{3} } \right)} \right] = 0 \\ & \Rightarrow a_{3} + a_{2} \zeta \: + a_{1} \zeta \:^{2} + \zeta \:^{3} - a_{1} \phi \:^{2} - 3\zeta \:\phi \:^{2} + i\left( {a_{2} \phi \: + 2a_{1} \phi \: + 3\zeta \:^{2} \phi \: - \phi \:^{3} } \right) \\ & \quad + e^{{ - \tau \:\zeta \:}} \left[ {\left( {h_{2} \phi \: + 2h_{1} \zeta \:\phi \:} \right)sin\:\tau \:\phi \: + \left( {h_{3} + h_{2} \zeta \: + h_{1} \zeta \:^{2} - h_{1} \phi \:^{2} } \right){\mathrm{cos}}\tau \:\phi \:} \right] \\ & \quad + e^{{ - \tau \:\zeta \:}} \left[ {\left( {h_{1} \phi \:^{2} - h_{3} - h_{2} \zeta \: - h_{1} \zeta \:^{2} } \right)sin\:\tau \:\phi \: + \left( {h_{2} \phi \: + 2h_{1} \zeta \:\phi \:} \right){\mathrm{cos}}\tau \:\phi \:} \right] = 0. \\ \end{aligned}$$

Assuming, we let $\:\zeta\:\left({\tau\:}_{c}\right)=0$ for some $\:{\tau\:}_{c}>0$ and $\:\zeta\:\left(\tau\:\right)<0\:for\:0\le\:\tau\:\le\:{\tau\:}_{c},$ then the steady state $\:{E}^{*}$ may loss stability at $\:\tau\:={\tau\:}_{c}\:or\:\lambda\:\left({\tau\:}_{c}\right)=i\phi\:\left({\tau\:}_{c}\right).$ In fact $\:i\phi\:$ is a root of Equ (24) if and only if we take terms of $\:\phi\:$ only and the constants.

27 $$\:-{i\phi\:}^{3}-{{a}_{1}\phi\:}^{2}+{a}_{2}i\phi\:+{a}_{3}+\left(-{h}_{1}{\phi\:}^{2}+{h}_{2}i\phi\:+{h}_{3}\right)\left(\mathrm{cos}{\tau\:}_{1}\phi\:-isin\:{\tau\:}_{1}\phi\:\right)=0$$

In order for Equ (25) to hold, both the real and imaginary parts must be zero. By equating real parts and imaginary parts of the right side of Equ (25) to zero, we have the Equs;

28 $$\:\left.\begin{array}{c}{h}_{2}\phi\:sin\:\tau\:\phi\:+\left({h}_{3}-{h}_{1}{\phi\:}^{2}\right)\mathrm{cos}\tau\:\phi\:={{a}_{1}\phi\:}^{2}-{a}_{3}\\\:{h}_{2}i\phi\:\mathrm{cos}\tau\:\phi\:-\left({h}_{3}-{h}_{1}{\phi\:}^{2}\right)isin\:\tau\:\phi\:={i\phi\:}^{3}-{a}_{2}i\phi\:\end{array}\right\}$$

Squaring both sides of (18) one obtains;

29 $$\:\left.\begin{array}{c}{h}_{2}^{2}{\phi\:}^{2}{sin}^{2}\tau\:\phi\:+\left({h}_{3}^{2}-2{h}_{1}{h}_{3}{\phi\:}^{2}+{h}_{1}^{2}{\phi\:}^{4}\right){cos}^{2}\tau\:\phi\:=\:{a}_{1}^{2}{\phi\:}^{4}-2{a}_{1}{a}_{3}{\phi\:}^{2}+{a}_{3}^{2}\\\:{h}_{2}^{2}{\phi\:}^{2}{cos}^{2}\tau\:\phi\:+\left({h}_{3}^{2}-2{h}_{1}{h}_{3}{\phi\:}^{2}+{h}_{1}^{2}{\phi\:}^{4}\right){sin}^{2}\tau\:\phi\:=\:{\phi\:}^{6}-2{a}_{2}{\phi\:}^{4}+{a}_{2}^{2}{\phi\:}^{2}\end{array}\right\}$$

Adding up the squares of (27) we have

$$\begin{aligned} & h_{2}^{2} \phi \:^{2} \left( {sin^{2} \tau \:\phi \: + cos^{2} \tau \:\phi \:} \right) + \left[ {\left( {h_{3}^{2} - 2h_{1} h_{3} \phi \:^{2} + h_{1}^{2} \phi \:^{4} } \right)\left( {sin^{2} \tau \:\phi \: + cos^{2} \tau \:\phi \:} \right)} \right] \\ & = a_{1}^{2} \phi \:^{4} - 2a_{1} d_{3} \phi \:^{2} + a_{3}^{2} + \phi \:^{6} - 2a_{2} \phi \:^{4} + a_{2}^{2} \phi \:^{2} \\ \end{aligned}$$

But $\:{sin}^{2}\tau\:\phi\:+{cos}^{2}\tau\:\phi\:=1,$ then we have;

30 $$\begin{aligned} & h_{2}^{2} \phi \:^{2} + h_{3}^{2} - 2h_{1} h_{3} \phi \:^{2} + h_{1}^{2} \phi \:^{4} = a_{1}^{2} \phi \:^{4} - 2a_{1} a_{3} \phi \:^{2} + a_{3}^{2} + \phi \:^{6} - 2a_{2} \phi \:^{4} + a_{2}^{2} \phi \:^{2} \\ & \Rightarrow {\mathcal{Q}}\left( {\phi \:} \right) = \phi \:^{6} + \left( {a_{1}^{2} - 2a_{2} - h_{1}^{2} } \right)\phi \:^{4} + \left( {a_{2}^{2} - 2a_{1} a_{3} + 2h_{1} h_{3} - h_{2}^{2} } \right)\phi \:^{2} + a_{3}^{2} - h_{3}^{2} = 0 \\ \end{aligned}$$

By squaring Equ (26) and subsequently adding it to Equ (27), two key outcomes become apparent. First, the trigonometric components cancel out, leading to a polynomial expression that no longer contains the delay parameter $\:\tau\:$. Second, the resulting expression is an even-degree polynomial. This follows from the fact that squaring either an even or an odd function always produces an even function, and consequently, the polynomial obtained possesses only even powers. that is $\:\mathcal{h}{\left(-\phi\:\right)}^{2}={\left(\pm\:\mathcal{h}\left(\phi\:\right)\right)}^{2}=\mathcal{h}{\left(\phi\:\right)}^{2}$.

For further simplification of (28), we define a new variable by letting;

$$\:\left(\mathcal{g}\equiv\:{\phi\:}^{2},\:{\mathcal{k}}_{1}\equiv\:{a}_{1}^{2}-2{a}_{2}-{h}_{1}^{2},\:\:\:\:{\mathcal{k}}_{2}\equiv\:{a}_{2}^{2}-2{a}_{1}{a}_{3}+2{h}_{1}{h}_{3}-{h}_{2}^{2},\:{\mathcal{k}}_{3}\equiv\:{a}_{3}^{2}-{h}_{3}^{2}\right)\in\:\mathbb{R}$$

Then we have Equ (28) rewritten as

31 $$\:\mathcal{N}\left(\mathcal{g}\right)={\mathcal{g}}^{3}+{\mathcal{k}}_{1}{\mathcal{g}}^{2}+{\mathcal{k}}_{2}\mathcal{g}+{\mathcal{k}}_{1}=0$$

where $\:\mathcal{N}$ is a polynomial. Since our analysis is restricted to values $\:\varphi\:\in\:\mathbb{R}$, it follows that if all real roots of the polynomial $\:\mathcal{N}$ are non-positive, then Equ s (26) cannot admit a simultaneous solution. On the other hand, the existence of a non-negative real root $\:g\:$of $\:\mathcal{N}$ guarantees the presence of a corresponding delay $\:\tau\:$ and frequency $\:\varphi\:$ that satisfy Equ (26).

#### Lemma 1

*Suppose that Equ (29) possesses only negative real roots. Then, for any *$\:\tau\:>0,$* all eigenvalues of Equ (23) have strictly negative real parts (see*^49^.

#### *Proof*

If all real roots of Equ (29) are negative, then no real value of $\:\varphi\:$ can satisfy Equ (20). Consequently, Equ (25) admits no real solution $\:{\sigma\:}_{1}$. This implies that, for every real $\:\varphi\:$, the complex number $\:i\varphi\:$cannot be a root of Equ (23). As a result, there exists no critical delay $\:{\tau\:}_{c}$such that $\:\lambda\:\left({\tau\:}_{c}\right)=i\varphi\:\left({\tau\:}_{c}\right)$ solves Equ (24). At $\:\tau\:=0$, the roots of Equ (23) satisfy $\:\mathrm{R}\mathrm{e}\left(\lambda\:\right(0\left)\right)<0$. Since the real parts of the roots depend continuously on the delay parameter $\:\tau\:$, it follows that the real parts remain negative for all $\:\tau\:>0$. Hence, all solutions of Equ (23) are stable for any admissible delay. Moreover, since Equ (29) is an odd-degree polynomial (specifically cubic), it necessarily admits at least one real root. The appearance of a simple positive real root would require the existence of two positive real roots, which does not occur under the stated assumptions. To characterize the conditions under which Equ (29) admits positive real roots, or alternatively none at all, we employ the Sturm sequence associated with polynomial (29), denoted by:

$$\:{\mathcal{N}}_{0}\left(\mathcal{g}\right)=\mathcal{N}\left(\mathcal{g}\right);\:\:\:\:\:\:\:\:\:\:{{\mathcal{N}}_{1}\left(\mathcal{g}\right)=\mathcal{N}}^{{\prime\:}}\left(\mathcal{g}\right).$$

Therefore, $$\:\:\:\:\:\:\:\:\:\:\:\:\:\:\:\:\:\:\:\:\:\:\:\:\:\:{\mathcal{N}}_{0}\left(\mathcal{g}\right)=\mathcal{N}\left(\mathcal{g}\right)={\mathcal{g}}^{3}+{\mathcal{k}}_{1}{\mathcal{g}}^{2}+{\mathcal{k}}_{2}\mathcal{g}+{\mathcal{k}}_{3}=0\:\:\:\:\:\:\:\:\:\:\:\:\:\:\:\:\:\:\:\:\:\:\:\:\:\:\:\:\:\:\:\:\:\:\:\:$$

32 $$\:{\mathcal{N}}_{1}\left(\mathcal{p}\right)=\mathcal{N}\mathcal{{\prime\:}}\mathcal{\:}\left(\mathcal{p}\right)=3{\mathcal{g}}^{2}+{2\mathcal{k}}_{1}\mathcal{g}+{\mathcal{k}}_{2}=0$$

The roots of (33) are

$$\:{\mathcal{g}}_{1}=\frac{-{\mathcal{k}}_{1}+\sqrt{{{\mathcal{k}}_{1}}^{2}-3{\mathcal{k}}_{2}}}{3},\:{\mathcal{g}}_{2}=\frac{-{\mathcal{k}}_{1}-\sqrt{{{\mathcal{k}}_{1}}^{2}-3{\mathcal{k}}_{2}}}{3}.$$

One of these is positive if $\:{\mathcal{k}}_{1}<0\:or\:{\mathcal{k}}_{1}>0\:and\:{\mathcal{k}}_{2}<0,$ so either $\:{\mathcal{k}}_{1}\:or\:{\mathcal{k}}_{2}$ must be negative. So we have;

#### Theorem 1

*If *$\:{\mathcal{k}}_{1}>0,\:{\mathcal{k}}_{2}\ge\:0,$ *then the delay equilibrium is asymptotically stable for all delay*$\:\tau\:\ge\:0.$

#### *Proof*

We will introduce the Hopf Bifurcation theorem Hassard et al.^40^, , as shall be discussed below.

To establish Hopf bifurcation at $\:\tau\:={\tau\:}_{c},$ we need to show that

33 $$\:\frac{d}{d\tau\:}\zeta\:\left(\tau\:\right)\ne\:0.$$

Now to ensure that Hopf bifurcation occurs, we provide conditions on the parameters. Suppose that Equ (28) has a positive root. We denote by $\:{\mathcal{g}}_{j},\:j\in\:\left\{1\right\}$ or $\:j\in\:\left\{1,\:2,\:3\right\}$ depending on the number of positive roots that Equ (28) has. Observe that Equ (30) has up to six roots i.e.,$\:\pm\:\sqrt{{\mathcal{g}}_{j}}\:for\:j=1,\:2,\:3.$

Notice that if the solution of Equ (23) exists, it is among these $\:\pm\:\sqrt{{\mathcal{p}}_{j}}\:for\:j=1,\:2,\:3$ and that if $\:\lambda\:=i$ is a root of Equ (23), $\:-i\phi\:$ will also be a root.

To Equ s (26) we substitute $\:{\phi\:}_{j}$ and then solve for $\:\tau\:$, which will then be of the form

$$\:{\tau\:}_{j}^{\left(n\right)}=\frac{1}{{\phi\:}_{j}}{\mathrm{sin}}^{-1}\left[\frac{{h}_{1}{\phi\:}_{j}^{5}+\left({a}_{1}{h}_{2}-{h}_{3}-{a}_{2}{h}_{1}\right){\phi\:}_{j}^{3}+\left({a}_{1}{h}_{3}-{a}_{3}{h}_{2}\right){\phi\:}_{j}}{{h}_{2}^{2}{\phi\:}_{j}^{2}+{\left({h}_{3}-{h}_{1}{\phi\:}_{j}^{2}\right)}^{2}}\right]+\left[\frac{2\pi\:\left(n-1\right)}{{\phi\:}_{j}}\right]$$

where$$\:j=0,\:1,\:2.$$.

if we multiply first sub Equ of (26) by $\:{h}_{2}{\phi\:}_{c}$ and the second sub Equ of (26) by $\:{h}_{3}-{h}_{1}{\phi\:}_{c}^{2}$ and also add the resulting product, we obtain

$$\begin{aligned} & h_{2}^{2} \phi \:_{c}^{2} {\mathrm{sin}}\phi \:_{c} \tau \:_{c} + h_{2} \phi \:_{c} \left( {h_{3} - h_{1} \phi \:_{c}^{2} } \right){\mathrm{cos}}\phi \:_{c} \tau \:_{c} = h_{2} \phi \:_{c} \left( {a_{1} \phi \:_{c}^{2} - a_{3} } \right) \\ & - h_{2} \phi \:_{c} \left( {h_{3} - h_{1} \phi \:_{c}^{2} } \right){\mathrm{cos}}\phi \:_{c} \tau \:_{c} + \left( {h_{3} - h_{1} \phi \:_{c}^{2} } \right)^{2} {\mathrm{sin}}\phi \:_{c} \tau \:_{c} = - \left( {h_{3} - h_{1} \phi \:_{c}^{2} } \right)\left( {\phi \:_{c}^{3} - a_{2} \phi \:_{c} } \right) \\ & \quad \Rightarrow \left[ {h_{2}^{2} \phi \:_{c}^{2} + \left( {h_{3} - h_{1} \phi \:_{c}^{2} } \right)^{2} } \right]{\mathrm{sin}}\phi \:_{c} \tau \:_{c} = h_{2} \phi \:_{c} \left( {a_{1} \phi \:_{c}^{2} - a_{3} } \right) - \left( {h_{3} - h_{1} \phi \:_{c}^{2} } \right)\left( {\phi \:_{c}^{3} - a_{2} \phi \:_{c} } \right) \\ & {\mathrm{sin}}\sigma \:_{c} \tau \:_{c} = \frac{{h_{2} \phi \:_{c} \left( {a_{1} \phi \:_{c}^{2} - a_{3} } \right) - \left( {h_{3} - h_{1} \phi \:_{c}^{2} } \right)\left( {\phi \:_{c}^{3} - a_{2} \phi \:_{c} } \right)}}{{\left[ {h_{2}^{2} \phi \:_{c}^{2} + \left( {h_{3} - h_{1} \phi \:_{c}^{2} } \right)^{2} } \right]}} \\ & \tau \:_{c} = \frac{1}{{\phi \:_{j} }}{\mathrm{sin}}^{{ - 1}} \left[ {\frac{{h_{1} \phi \:_{j}^{5} + \left( {a_{1} h_{2} - h_{3} - a_{2} h_{1} } \right)\phi \:_{j}^{2} + \left( {a_{2} h_{3} - a_{3} h_{2} } \right)\phi \:_{j} }}{{h_{2}^{2} \phi \:_{j}^{2} + \left( {h_{3} - h_{1} \phi \:^{2} } \right)^{2} }}} \right] + \left[ {\frac{{2\pi \:\left( {n - 1} \right)}}{{\phi \:_{j} }}} \right] \\ & \tau \:_{j}^{{\left( n \right)}} = \frac{1}{{\phi \:_{j} }}{\mathrm{sin}}^{{ - 1}} \left[ {\frac{{h_{1} \phi \:_{j}^{5} + \left( {a_{1} h_{2} - h_{3} - a_{2} h_{1} } \right)\phi \:_{j}^{2} + \left( {a_{2} h_{3} - a_{3} h_{2} } \right)\phi \:_{j} }}{{h_{2}^{2} \phi \:_{j}^{2} + \left( {h_{3} - h_{1} \phi \:^{2} } \right)^{2} }}} \right] + \left[ {\frac{{2\pi \:\left( {n - 1} \right)}}{{\phi \:_{j} }}} \right]. \\ \end{aligned}$$

Now let $\:{\tau\:}_{c}$ be the smallest of such $\:\tau\:$ for which $\:{\zeta\:}_{c}\left({\tau\:}_{c}\right)=0.$.

Therefore,

34 $$\tau _{c} = \tau _{{j_{c} }}^{{\left( {n_{c} } \right)}} = \left. {\begin{array}{*{20}l} {{\mathrm{min}}\left\{ {\tau _{j}^{{\left( n \right)}} > 0,0 \le j \le 2,n \ge 1} \right\}} \hfill \\ {\zeta _{c} = \zeta _{{j_{c} }} } \hfill \\ \end{array} } \right\}$$

#### Theorem 2

*Let the critical time delay* $$\:{\tau\:}_{c}\:and\:{\sigma\:}_{c}$$ *be defined as (32) for the time delay* $$\:{\tau\:}_{1}$$ *and suppose that* $$\:{3\phi\:}_{c}^{6}+{2{\mathcal{k}}_{1}\phi\:}_{c}^{4}+{{\mathcal{k}}_{2}\phi\:}_{c}^{2}\ne\:0$$ *then the system of delay differential Equ (7) exhibits the Hopf bifurcation at the persistent inflammation equilibrium* $$\:\:{E}^{**}$$

#### *Proof*

To establish Hopf bifurcation, we show that

35 $$\:\frac{d\zeta\:\left(\tau\:\right)}{d\tau\:}{|}_{\tau\:{=\tau\:}_{c}}\ne\:0$$

and this guarantees that Hopf bifurcation occurs. For (32) to hold, both the real and imaginary parts of (16) must be zero. So we get the pair of Eq. 

36 $$\:{e}^{-\tau\:\zeta\:}\left[\left({h}_{2}\phi\:+2{h}_{1}\zeta\:\phi\:\right)\mathrm{sin}\tau\:\zeta\:+\left({h}_{3}+{h}_{2}\zeta\:-{h}_{1}{\zeta\:}^{2}-{h}_{1}{\phi\:}^{2}\right)\mathrm{cos}\tau\:\phi\:\right]=\left({a}_{1}+3\zeta\:\right){\phi\:}^{2}-{a}_{3}-{a}_{2}\zeta\:-{a}_{1}{\zeta\:}^{2}-{\zeta\:}^{3}$$

37 $$\:{e}^{-\tau\:\phi\:}\left[\left({h}_{1}{\phi\:}^{2}-{h}_{3}-{h}_{2}\zeta\:-{h}_{1}{\zeta\:}^{2}\right)\mathrm{sin}\phi\:+\left({h}_{2}\phi\:+2{h}_{1}\zeta\:\right)\mathrm{cos}\tau\:\phi\:\right]={\phi\:}^{3}-{a}_{2}\phi\:-2{a}_{1}\zeta\:\phi\:-3{\zeta\:}^{2}\phi\:$$

Solving equs. (34) and (35) simultaneously, we obtain

$$\begin{aligned} & \frac{{\begin{array}{*{20}c} {\left[ {3\phi \:_{c}^{6} + 2a_{1}^{2} \phi \:_{c}^{4} - 4a_{2} \phi \:_{c}^{4} - 2h_{1}^{2} \phi \:_{c}^{4} + a_{2}^{2} \phi \:_{c}^{2} - 2a_{1} a_{3} \phi \:_{c}^{2} + 2h_{1} h_{3} \phi \:_{c}^{2} - h_{2}^{2} \phi \:_{c}^{2} } \right]} \\ {\:\left[ {h_{2}^{2} \phi \:_{c}^{2} + h_{3}^{2} - 2h_{1} h_{3} \phi \:_{c}^{2} + h_{1}^{2} \phi \:_{c}^{4} } \right]} \\ \end{array} }}{{\left[ {h_{2}^{2} \phi \:_{c}^{2} + h_{3}^{2} - 2h_{1} h_{3} \phi \:_{c}^{2} + h_{1}^{2} \phi \:_{c}^{4} } \right]\left[ {{\mathcal{B}}_{1}^{2} + {\mathcal{B}}_{2}^{2} } \right]}} \\ & = \frac{{3\phi \:_{c}^{6} + 2\left( {a_{1}^{2} - 2a_{2} - h_{1}^{2} } \right)\phi \:_{c}^{4} + \left( {a_{2}^{2} - 2a_{1} a_{3} + 2h_{1} h_{3} - h_{2}^{2} } \right)\phi \:_{c}^{2} }}{{{\mathcal{B}}_{1}^{2} + {\mathcal{B}}_{2}^{2} }} \\ \end{aligned}$$

If $\:{a}_{1}^{2}-2{a}_{2}-{h}_{1}^{2}={\mathcal{k}}_{1}\:and\:{a}_{2}^{2}-2{a}_{1}{a}_{3}+2{h}_{1}{h}_{3}-{h}_{2}^{2}={\mathcal{k}}_{2},$ then we have;

38 $$\left. {\frac{{d\zeta }}{{d\tau }}} \right|_{{\tau = \tau _{c} }} = \frac{{3\phi _{c}^{6} + 2{\mathcal{h}}_{1} \phi _{c}^{4} + {\mathcal{h}}_{2} \phi _{c}^{2} }}{{{\mathcal{B}}_{1}^{2} + {\mathcal{B}}_{2}^{2} }} \ne 0$$

Therefore, we conclude that Hopf bifurcation occurs when $\:\tau\:$ passes through the critical value, which is assumed to be $\:{\tau\:}_{c}.$

## Simulation result and discussion

### Fractional-order derivative for TKR recovery dynamics

In this subsection, we discuss the experimental outcome of the knee implant fractional delay equation model with variable order differential operator used for the inflammation given in Equ (7). We assigned different fractional orders to the variables, to better capture their unique biological behaviors, interactions, and responses to rehabilitations and mechanical function, leading to more accurate predictions and insights into knee implant surgery recovery dynamics. By using different fractional orders, the model can more precisely represent the heterogeneous dynamics of knee implant surgery recovery dynamics, inflammation and mechanical function of the implant, reflecting their distinct biological and environmental interactions. We analyse the dynamical activities of the proposed model for the variation of the fractional-order derivative $\:\mathcal{q},$ and variations of the inflammation level in the absence and presence of delay. In Table 2, we provide the parameter values that are used in the numerical simulation, and the initial conditions are taken as $\:R\left(0\right)=0.5,\:I\left(0\right)=0.2,\:M\left(0\right)=0.6.$.

### Results and discussions

**Fig. 2:**
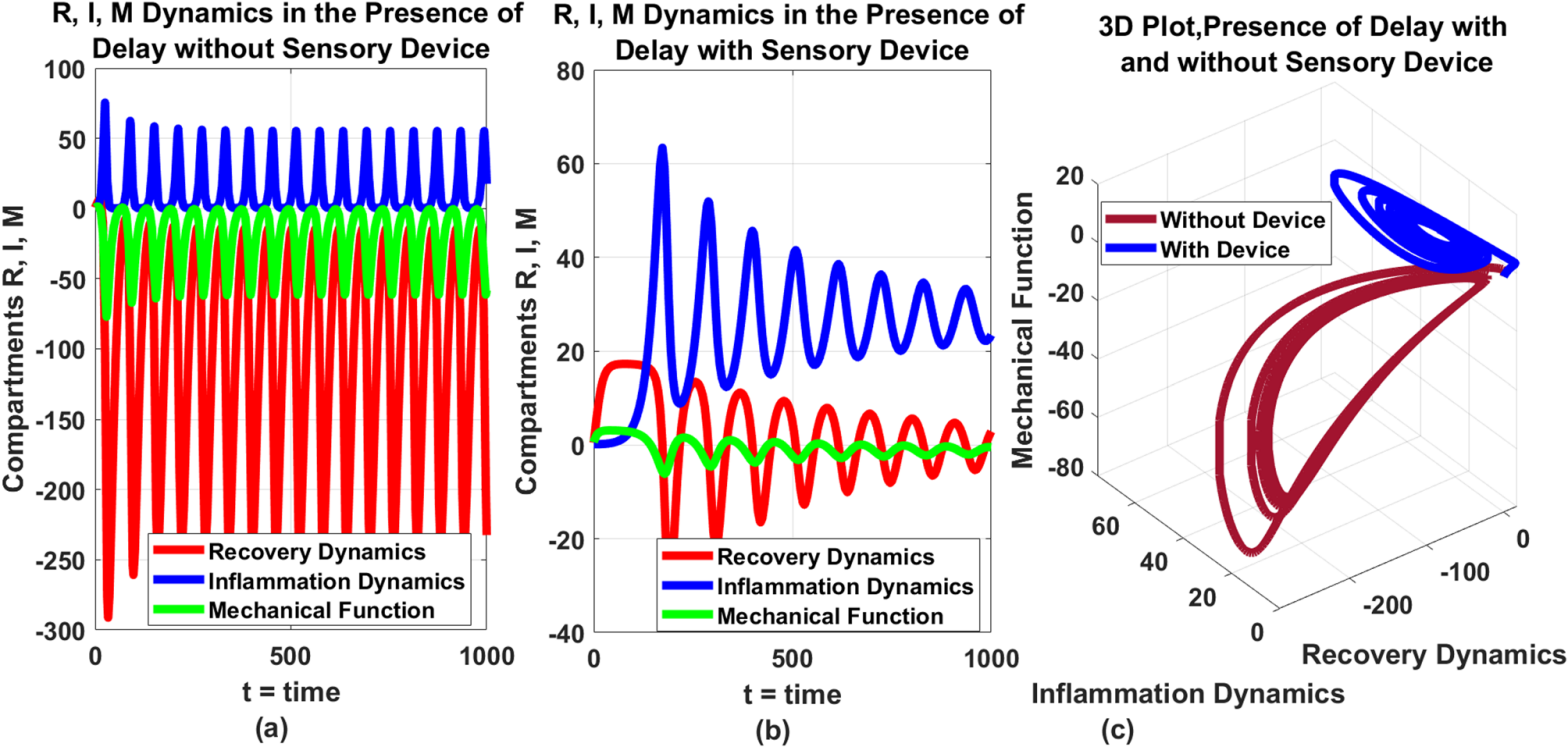
(**a**–**c**) Numerical simulation of Equ (7) where $\:\alpha\:=0.9,\:\beta\:=0.8,\:\gamma\:=0.001,\:\delta\:=0.2,\:\mu\:=\left(0.005-0.5\right),\:\eta\:=\left(0.0004-0.002\right),\:\rho\:=0.8,\:\pi\:=0.04,\:\sigma\:=0.25,\:\tau\:=5,\xi\:=0.09,\:\vartheta\:=0.05$ with high inflammation in the presence of delay.

**Fig. 3:**
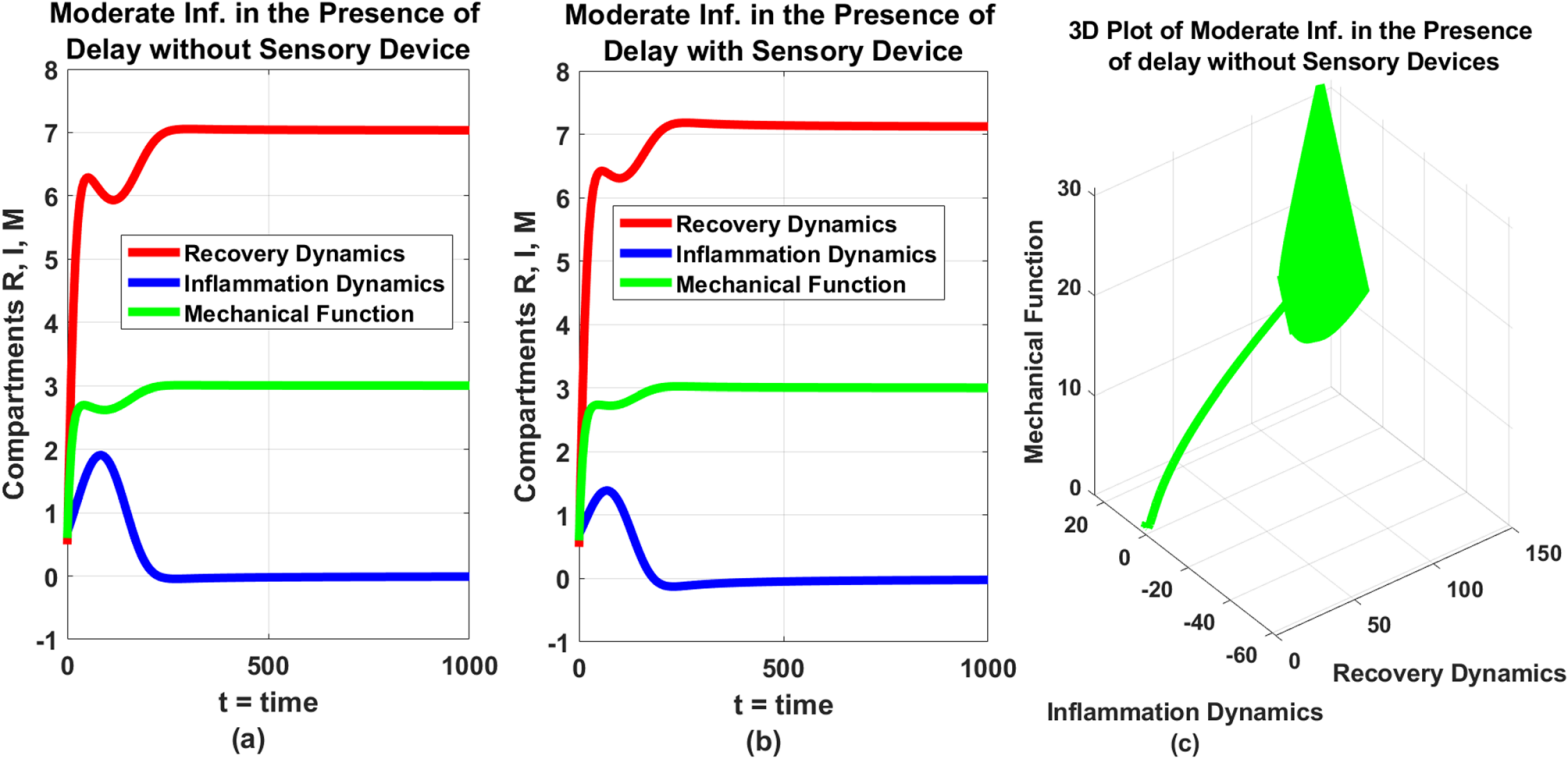
(**a**–**c**) Numerical simulation of Equ (7) where $\:\alpha\:=0.5,\:\beta\:=0.3,\:\gamma\:=0.0001,\:\delta\:=0.2,\:\mu\:=0.05,\:\eta\:=0.002,\:\rho\:=0.6,\:\pi\:=0.04,\:\sigma\:=0.2,\:\tau\:=5,\:\xi\:=0.09,\:\vartheta\:=0.05$ with moderate inflammation in the presence of delay.

Figures 2 and 3 show the normalised time evolution of recovery $\:R\left(t\right)$, inflammation $\:I\left(t\right)$, and mechanical function $\:M\left(t\right)\:$under delayed feedback, both with and without sensory-device–assisted monitoring. In the high-inflammation regime (Fig. 2), the absence of sensory feedback leads to large-amplitude oscillations driven by delay-induced instability. This results in degraded mechanical performance. The negative values of the inflammation variable indicate suppression below the baseline reference rather than pathological inflammation. Incorporation of sensory monitoring significantly attenuates oscillations, stabilises recovery, and improves mechanical function, as further illustrated by the compact phase trajectories in the 3D plots.

**Fig. 4:**
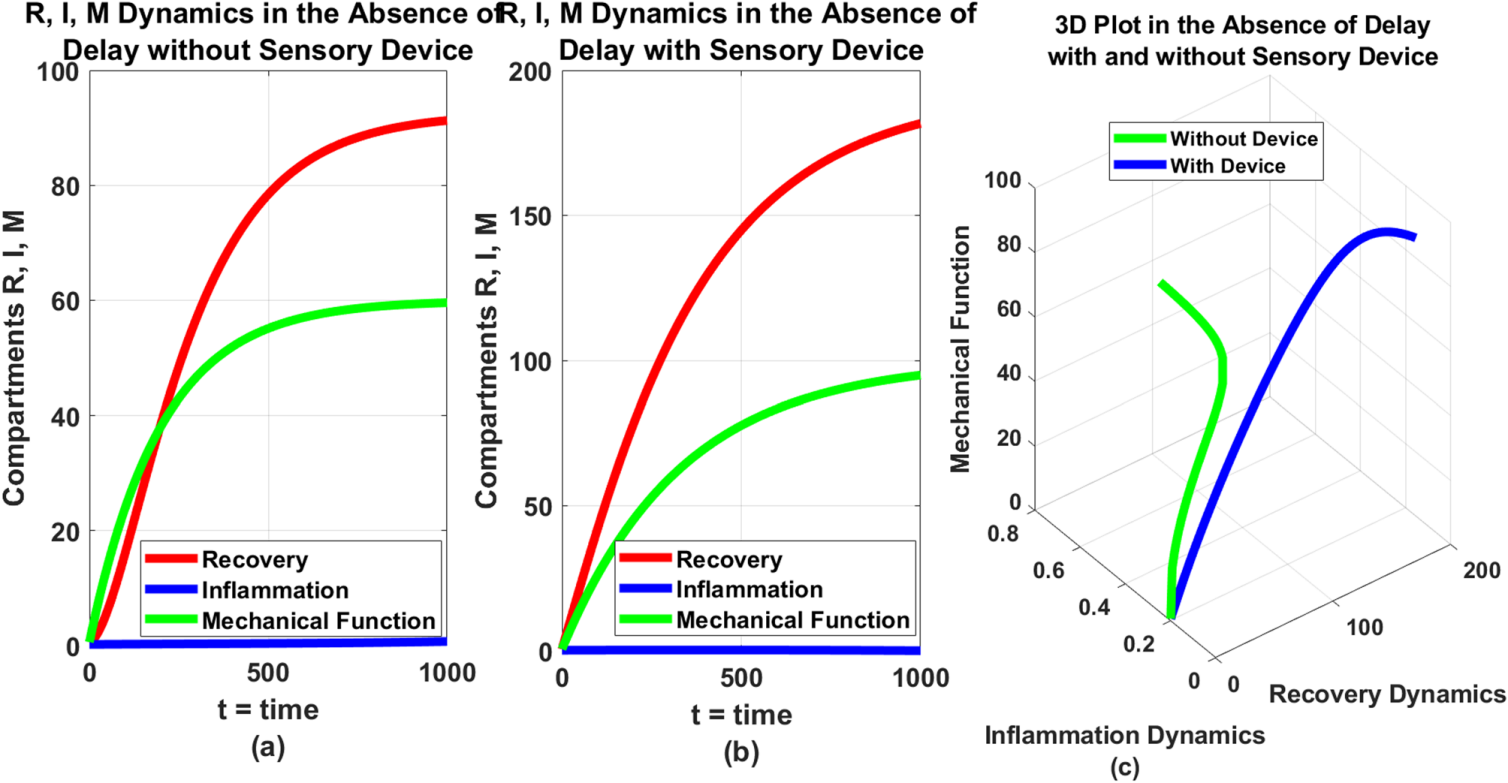
(**a**–**c**) Numerical simulation of Eq. (9) where $\:\alpha\:=0.05,\:\beta\:=0.03,\:\gamma\:=0.005,\:\delta\:=0.02,\:\mu\:=0.005,\:\eta\:=0.00002,\:\rho\:=0.6,\:\pi\:=0.004,\:\sigma\:=0.01,\xi\:=0.09,\:\vartheta\:=0.05,\:\tau\:=0,$ with low inflammation in the absence of delay.

Under moderate inflammation (Fig. 3), the system converges toward equilibrium even without sensory assistance, though with slower transients. Sensory monitoring accelerates convergence and enhances stability, yielding smoother recovery and improved functional outcomes. Generally, the simulations highlight the stabilising role of enhanced monitoring in mitigating delay-driven instability and promoting sustained recovery. Figure 4 illustrates the system behavior under low inflammation and the absence of delay.

In Fig. 4(a), without sensory assistance, recovery and mechanical function increase monotonically and converge to stable steady states, while inflammation remains close to its baseline reference, indicating a favourable and stable recovery environment. When sensory monitoring is incorporated (Fig. 4(b)), both recovery and mechanical function attain higher asymptotic levels, demonstrating that sensing feedback enhances outcomes even under already optimal conditions. The corresponding 3D phase trajectories in Fig. 4(c) show smooth, monotonic convergence toward equilibrium, with the sensory-assisted case exhibiting superior performance, confirming system stability and predictability in the absence of inflammatory and delay effects.

**Fig. 5:**
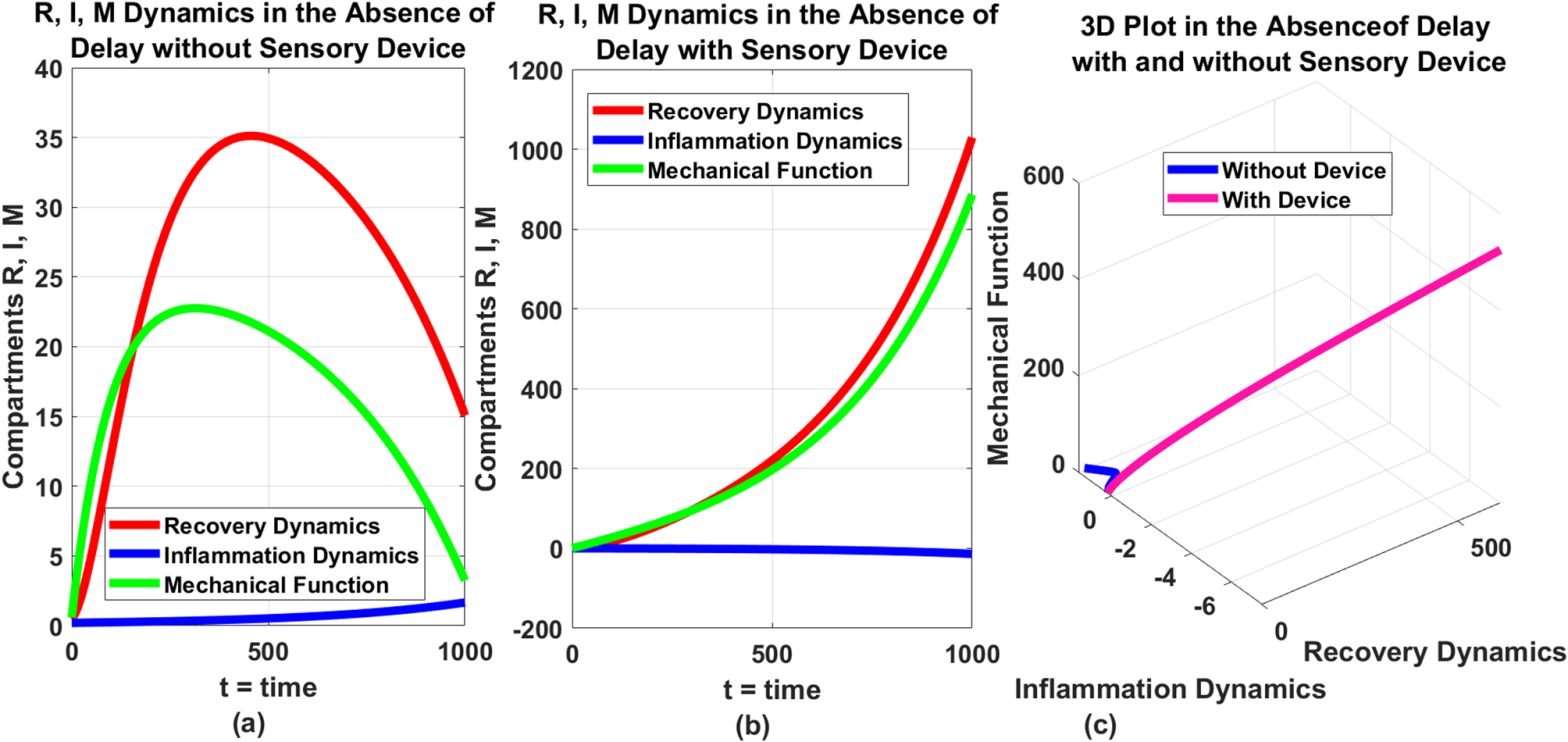
(**a**–**c**) Numerical simulation of Eq. (9) where $\:\alpha\:=0.05,\:\beta\:=0.03,\:\gamma\:=0.01,\:\delta\:=0.02,\:\mu\:=0.005,\:\eta\:=0.00002,\:\rho\:=0.6,\:\pi\:=0.4,\:\sigma\:=0.01,\xi\:=0.09,\:\vartheta\:=0.05,\:\tau\:=0$ with high inflammation in the absence of delay.

Figure 5 examines high inflammation without delay. In the absence of sensory input (Fig. 5(a)), recovery and mechanical function display transient growth followed by decline, reflecting the sustained detrimental influence of inflammation. In contrast, Fig. 5(b) shows that sensory monitoring stabilises recovery and mechanical performance while suppressing inflammation, indicating improved regulation of pathological activity. The 3D phase plot in Fig. 5(c) highlights an inverse relationship between inflammation and mechanical function, reinforcing the adverse impact of persistent inflammation and the compensatory role of sensing feedback in restoring functional stability.

**Fig. 6:**
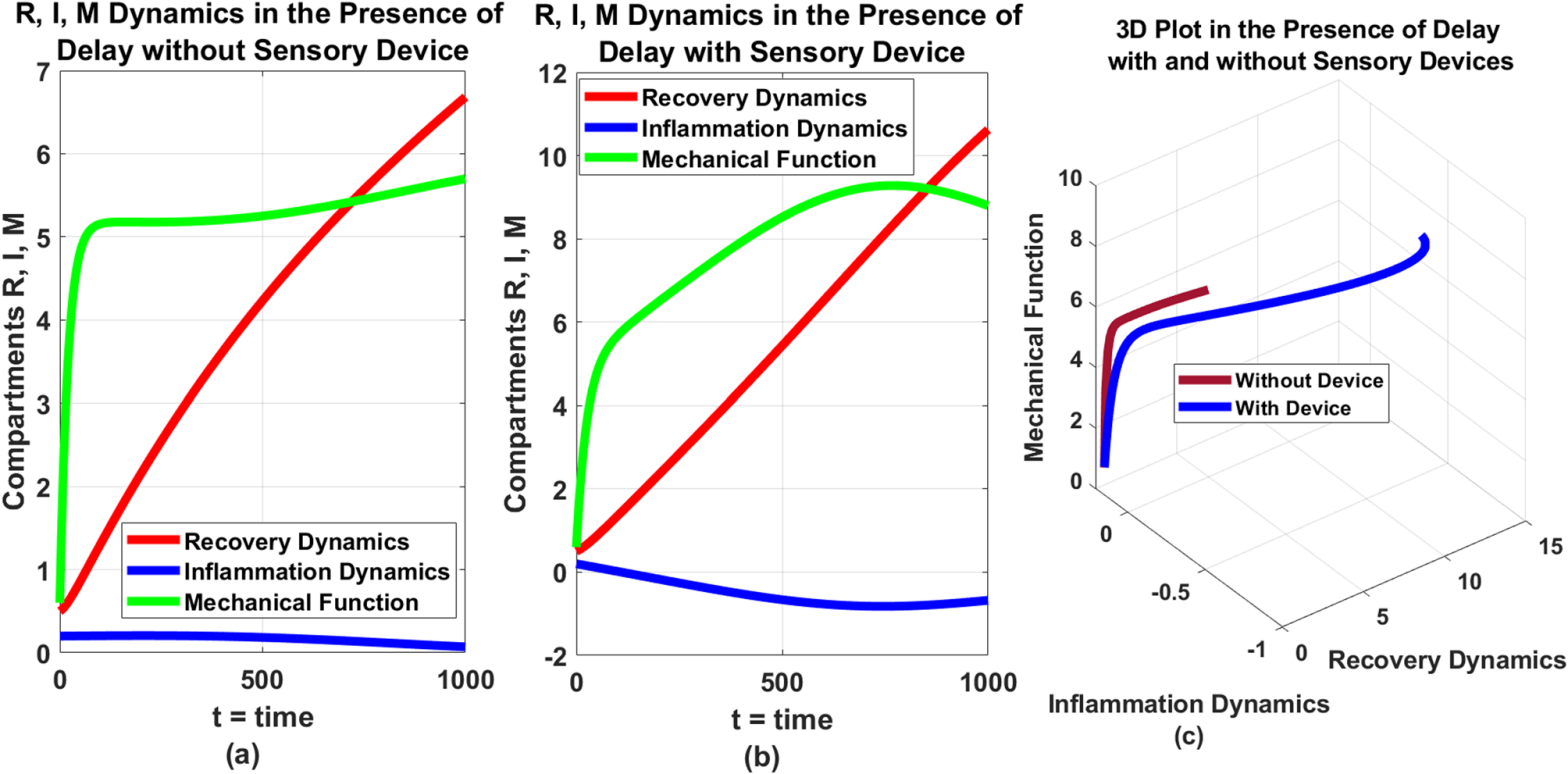
(**a**–**c**) Numerical simulation of Eq. (9) where $\:\alpha\:=0.005,\:\beta\:=0.003,\:\gamma\:=0.000001,\:\delta\:=0.002,\:\mu\:=0.0005,\:\eta\:=0.00002,\:\rho\:=0.6,\:\pi\:=0.4,\:\sigma\:=0.01,\xi\:=0.09,\:\vartheta\:=0.05,\:\tau\:=5$ with low inflammation in the presence of delay.

Figure 6 considers low inflammation in the presence of delay.

**Fig. 7:**
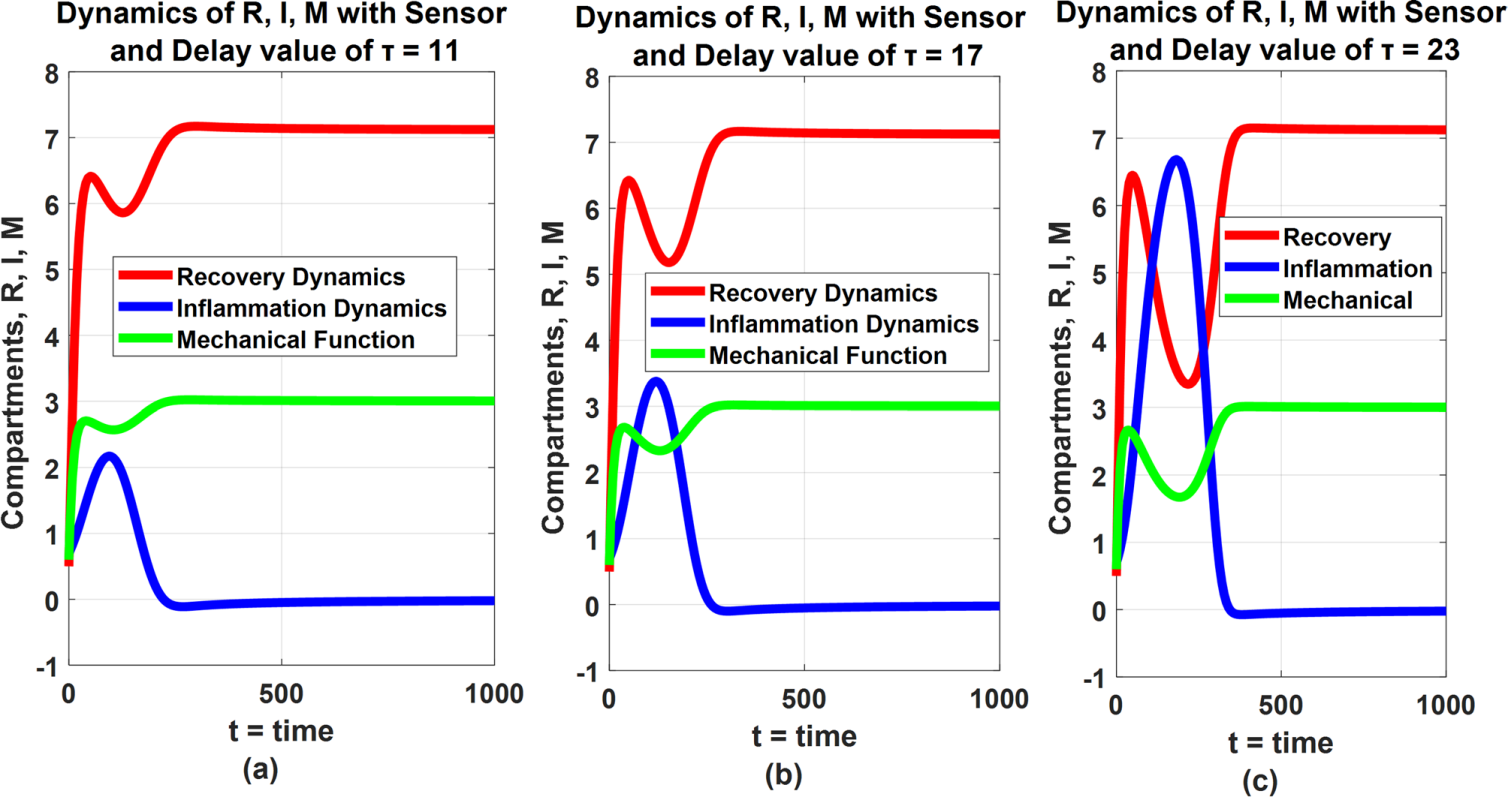
(**a**–**c**) Dynamics of the knee implant recovery, inflammation and mechanical function as in fractional order delay differential Eq. model (7) in the presence of sensing devices with $\:\tau\:=11,\:17,\:and\:23.$.

Without sensory assistance (Fig. 6(a)), recovery and mechanical function increase gradually, while inflammation remains controlled but persistent. With sensory integration (Fig. 6(b)), recovery and mechanical function converge more rapidly and to higher steady states, while inflammation is more effectively suppressed, occasionally falling below baseline, indicating strong regulatory control. The 3D trajectory in Fig. 6(c) further confirms that sensing feedback mitigates delay-induced effects and promotes system stabilisation. Figure 7 explores the influence of increasing delay values ($\:\tau\:=\mathrm{11,17,23}$) under sensory-assisted conditions. At lower delay ($\:\tau\:=11$), all compartments exhibit mild transient oscillations before stabilising.

As delay increases ($\:\tau\:=17$), oscillations become more pronounced, and convergence slows. At higher delay ($\:\tau\:=23$), recovery and inflammation show larger transient oscillations and delayed stabilisation, although long-term equilibrium is still achieved. These results indicate that increased delay weakens immediate feedback synchronisation, while timely sensing and reduced delay enhance stability and recovery efficiency. Consequently, the simulations demonstrate that sensory-device–assisted feedback consistently improves recovery and mechanical outcomes, suppresses inflammation, and mitigates delay-induced instability, with effectiveness diminishing as physiological or feedback delays increase.

One useful application of our model is in modern smart hospitals. In this setting, knee implant recovery is transformed from a manual, episodic process into a continuous, AI-driven, patient-centric system. The approach enhances the healing process, reduces complications, and enables personalised rehabilitation.

From the simulation results above, Table 4 summarises the percentage improvements in recovery $\:R\left(t\right)$, inflammation $\:I\left(t\right)$, and mechanical function $\:M\left(t\right)$among knee implant patients using sensing devices under both delayed and non-delayed conditions. In the presence of delays, sensing devices provided moderate to high improvements across all three outcomes, with particularly strong benefits for inflammation control. In the absence of delays, the impact of sensing devices was even more pronounced, showing near-maximal enhancement in recovery, inflammation reduction, and mechanical function. These results underscore the critical role of sensing devices in optimising postoperative outcomes, especially when physiological or systemic delays are minimal.

**Table 4:** Percentage improvement in Recovery $\:R\left(t\right)$, Inflammation $\:I\left(t\right)$ and Mechanical Function $\:M\left(t\right)$ of Knee implant surgery, Patients with sensing devices in the presence and absence of delay.

$\:\mathrm{R}\left(\mathrm{t}\right)$ Improvement with Sensing Devices	$\:\mathrm{R}\left(\mathrm{t}\right)$ Improvement without Sensing Devices	$\:\mathrm{\%}$ Improvement in $\:\mathrm{R}\left(\mathrm{t}\right)$	$\:\mathrm{I}\left(\mathrm{t}\right)$ Improvement with Sensing Devices	$\:\mathrm{I}\left(\mathrm{t}\right)$ Improvement without Sensing Devices	$\:\mathrm{\%}$ Improvement in $\:\mathrm{I}\left(\mathrm{t}\right)$	$\:\mathrm{M}\left(\mathrm{t}\right)$ Improvement with Sensing Devices	$\:\mathrm{M}\left(\mathrm{t}\right)$ Improvement without Sensing Devices	$\:\mathrm{\%}$ Improvement in $\:\mathrm{M}\left(\mathrm{t}\right)$
Presence of Delay								
181.6766	91.2687	49.76	0.05732	0.71652	92.00	94.9239	59.5758	37.24
Absence of Delay								
1029.4759	15.0984	98.53	-13.8131	1.6628	100.00	886.7317	3.2598	99.63

### Summary of contributions

This study presents a physiologically informed fractional-order model for total knee replacement (TKR) recovery. The formulation in Sect.  2 captures non-local temporal effects from cumulative stress, strain, and biological signalling, while explicitly including delays associated with immune response and tissue regeneration. These features provide a more realistic representation of post-surgical rehabilitation dynamics. Rigorous mathematical analysis establishes solution existence and stability conditions around equilibrium states, ensuring analytical robustness and biological interpretability. The model embeds control parameters for therapy intensity, medication scheduling, and patient adherence, enabling personalised optimisation of recovery pathways. Numerical simulations, implemented via a fractional-order two-step Lagrange interpolation scheme, validate the theoretical findings and generate recovery trajectories consistent with clinical observations.

Table 4 quantifies the impact of the sensing device and delays on recovery $\:R\left(t\right)$, inflammation $\:I\left(t\right)$, and mechanical function $\:M\left(t\right)$. In the presence of delay, sensing devices improve recovery by ~ 50%, reduce inflammation by ~ 92%, and enhance mechanical function by ~ 37% compared to scenarios without sensing. When delays are absent, improvements are even more pronounced, with recovery increasing by ~ 99%, inflammation controlled by ~ 100%, and mechanical function improved by ~ 99%. These results highlight two key insights: (i) delays significantly limit the effectiveness of recovery, and (ii) patient-specific sensing devices dramatically enhance post-operative outcomes, especially in delayed physiological responses. Compared to existing TKR models that assume instantaneous biological responses, this framework captures long-term memory effects and immune–tissue response latency in a unified manner. By integrating fractional dynamics, biologically motivated delays, and explicit control mechanisms, the model supports patient-specific predictions, therapy optimisation, and rehabilitation planning. Overall, it provides a mathematically validated, clinically relevant framework that quantifies how monitoring, therapy timing, and inflammation control jointly influence long-term recovery outcomes. In Sect.  5.4, we shall present the implementation framework in detail.

To ensure clinical interpretability, it is important to note that the model variables $\:R\left(t\right)$, $\:I\left(t\right)$, and $\:M\left(t\right)$ are defined as normalised, dimensionless state variables referenced to baseline post-operative conditions, rather than absolute clinical measurements. In particular, inflammation $\:I\left(t\right)$is modelled as a deviation from baseline, where zero represents the reference inflammatory level immediately after surgery, positive values indicate elevated inflammation, and negative values correspond to reduced inflammation below baseline due to effective therapeutic intervention. Consequently, the negative inflammation values observed in the absence of delay reflect enhanced inflammatory control enabled by real-time sensing and feedback mechanisms in smart healthcare systems. Similarly, the large percentage improvements reported in Table 4 arise from relative comparisons between intervention scenarios within this normalised framework. These values should therefore be interpreted as indicators of the relative efficiency and effectiveness of sensing-device-assisted recovery pathways, rather than as absolute clinical percentage changes. Overall, the results highlight that minimising delays in smart healthcare infrastructures significantly enhances recovery, suppresses inflammation, and improves mechanical function following knee implant surgery.

### Practical IoT-enabled KRR intelligent monitoring system

**Fig. 8:**
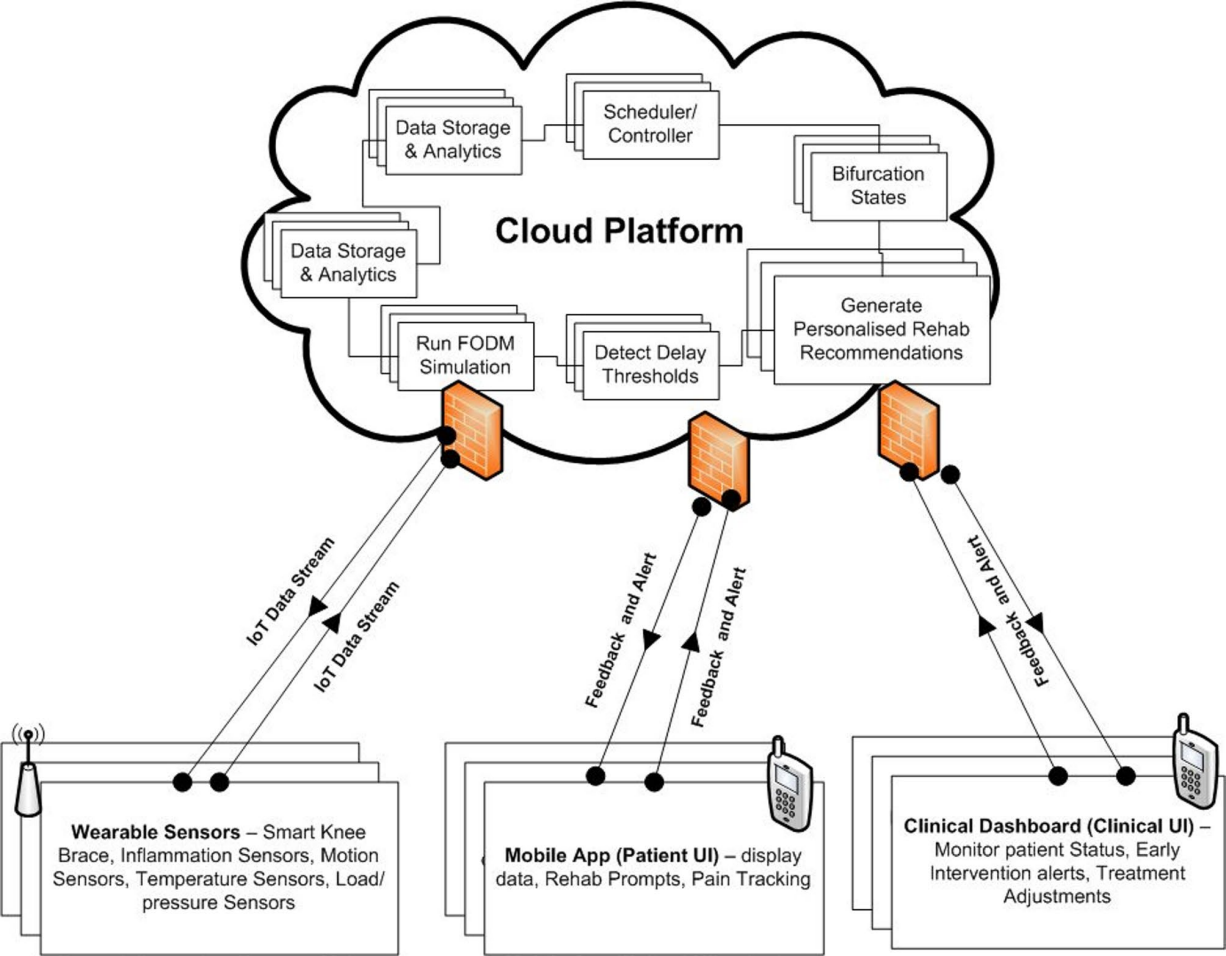
Smart IoT-powered framework for KRR. This captures FODCM and provides a control-oriented, real-time management architecture that integrates IoT sensing to optimise system performance.

In this use case, KRR system integrates IoT-enabled sensing to facilitate real-time physiological monitoring, personalised feedback, and adaptive clinical interventions (Fig. 8). Wearable sensors capture joint kinematics, pain thresholds, and indicators of inflammation, such as swelling or skin temperature. Concurrently, mechanical data acquisition systems track gait patterns, range of motion, and load distribution across knee implants. Biosensors measure biomarkers from skin, sweat, or metabolites to quantify inflammatory responses during the recovery process. The system generates real-time alerts for delayed progress or oscillatory recovery patterns, enabling timely clinician and patient interventions. IoT data streams are transmitted to cloud platforms and integrated with the FODCM framework to personalise rehabilitation protocols and optimise therapy outcomes^50^. Additionally, edge-based mobile applications enhance patient engagement by delivering reminders, educational content, progress tracking, and physiotherapy guidance. Figure 8 illustrates the overall framework, highlighting the synergy between wearable sensing, cloud analytics^51^, and feedback-driven rehabilitation, which collectively improve recovery efficiency, clinical outcomes, and patient adherence.

## Conclusion

This study presents a novel fractional-order delay model for knee implant recovery designed to support smart orthopaedic healthcare systems. The model employs fractional derivatives to capture memory and viscoelastic properties of musculoskeletal tissues, while delay terms represent physiological latencies associated with neuromuscular feedback, immune responses, and tissue remodelling. Simulation results demonstrate that lower fractional orders slow recovery due to stronger memory effects, and that prolonged delays can amplify instability under high-inflammatory conditions. Observed oscillatory responses further highlight the importance of early and sustained inflammation control during rehabilitation. Compared with traditional integer-order models, the proposed framework provides a more accurate and clinically relevant representation of post-operative recovery dynamics. By integrating biological memory, adaptive recovery behaviour, and delayed physiological responses, the model supports patient-specific predictions and rehabilitation planning. Validation against expected biomechanical patterns confirms the reliability of the approach and demonstrates its potential as a scalable tool for personalised, data-driven post-surgical care. Future work will focus on integrating AI-enabled agents with secure real-world patient datasets and smart health devices. Planned developments include deployment of IoT-enabled wearable platforms with virtual physiotherapy agents, enhancement of sensor accuracy, incorporation of inter-patient variability, and further refinement of physiological delay modelling.

## Supplementary Information

Below is the link to the electronic supplementary material.

Supplementary Material 1

Supplementary Material 2

## Data Availability

All data generated and analysed during this study were produced through computational simulation of the proposed fractional-order differential model. The simulated datasets supporting the findings are available from the corresponding author on reasonable request via GitHub: https://github.com/ken-cisco/Fractional-Order-Differential-Model-for-Knee-Implant-Recovery-in-Smart-Health-Infrastructures.git.
