# Supplementary material for: Fractional-order differential model for knee implant recovery in smart health infrastructures
Source: Sci Rep. 2026 Apr 17;16:17893. doi: 10.1038/s41598-026-48965-7 (PMC13249972; doi:10.1038/s41598-026-48965-7)
Supplement: Supplementary file 1 — Supplementary Material 1 [file 41598_2026_48965_MOESM1_ESM.docx]

**APPENDIX A: Proof of Theorem 2**

**Proof:** To establish Hopf bifurcation, we show that

$$\frac{d\zeta\left( \tau\right)}{d\tau}|_{\tau{=\tau}_{c}}\neq0 (33)$$

and this guarantees that Hopf bifurcation occurs. For (32) to hold, both the real and imaginary parts of (16) must be zero. So we get the pair of equations

$$e^{-\tau\zeta}\left[ \left( h_{2}\varphi+2h_{1}\zeta\varphi\right)\sin\tau\zeta+\left( h_{3}+h_{2}\zeta-h_{1}\zeta^{2}-h_{1}\varphi^{2} \right)\cos\tau\varphi\right]=\left( a_{1}+3\zeta\right)\varphi^{2}-a_{3}-a_{2}\zeta-a_{1}\zeta^{2}-\zeta^{3} \left( 34 \right)$$

$$e^{-\tau\varphi}\left[ \left( h_{1}\varphi^{2}-h_{3}-h_{2}\zeta-h_{1}\zeta^{2} \right)\sin\varphi+\left( h_{2}\varphi+2h_{1}\zeta\right)\cos\tau\varphi\right]=\varphi^{3}-a_{2}\varphi-2a_{1}\zeta\varphi-3\zeta^{2}\varphi(35)$$

From equation (34), we have that $e^{-\tau\zeta}=e^{0}$since $\tau\zeta=0$. Then $e^{0}=1,$ and selecting $\varphi$ from the equation we obtain

$$h_{2}\varphi\sin\tau\varphi+h_{3}\cos\tau\varphi-h_{1}\varphi^{2}\cos\tau\varphi=a_{1}\varphi^{2}-a_{3} (36)$$

We at this moment differentiate equation (28) with respect to $\tau$ after which we evaluate it at $\tau{=\tau}_{c}$ for which $\zeta\left( \tau_{c} \right)=0 and\varphi\left( \tau_{c} \right)=\varphi_{c}$ and get

$$\frac{d\varphi}{d\tau}|_{\tau{=\tau}_{c}}=h_{2}\sin\varphi_{c}\tau_{c}+\tau_{c}h_{2}\varphi_{c}\cos\varphi_{c}\tau_{c}-h_{3}\tau_{c}\sin\varphi_{c}\tau_{c}-2h_{1}\varphi_{c}\cos\varphi_{c}\tau_{c}+\tau_{c}h_{1}{\varphi_{c}}^{2}\sin\varphi_{c}\tau_{c}=2a_{1}\varphi_{c}$$

$$\Longrightarrow2a_{1}\varphi_{c}+\left( 2h_{1}\varphi_{c}-\tau_{c}h_{2}\varphi_{c} \right)\cos\varphi_{c}\tau_{c}+\left( h_{3}\tau_{c}-\tau_{c}h_{1}{\varphi_{c}}^{2}-h_{2} \right)\sin\varphi_{c}\tau_{c}=\mathcal{B}_{1}$$

Select $\zeta$from (34), differentiate with respect to $\tau$and evaluating at $\tau{=\tau}_{c}$ for which $\zeta\left( \tau_{c} \right)=0 and \varphi\left( \tau_{c} \right)=\varphi_{c},$ we then obtain

$$2h_{1}\zeta\varphi\sin\tau\varphi+h_{2}\zeta\cos\tau\varphi+h_{1}\zeta^{2}\cos\tau\varphi=3\zeta\varphi^{2} -a_{2}\zeta-a_{1}\zeta^{2}-\zeta^{3} (37)$$

$$\frac{d\zeta}{d\tau}|_{\tau{=\tau}_{c}}=2h_{1}\varphi\sin\varphi_{c}\tau_{c} +2\tau_{c}\zeta h_{1}\varphi_{c}\cos\varphi_{c}\tau_{c}+h_{2}\cos\varphi_{c}\tau_{c}-\tau_{c}h_{2}\zeta\sin\varphi_{c}\tau_{c}+2h_{1}\zeta\cos\varphi_{c}\tau_{c}$$

$$-h_{1}\zeta^{2}\tau_{c}\sin\varphi_{c}\tau_{c}=3{\varphi_{c}}^{2}-a_{2}-2a_{1}\zeta-3\zeta^{2}$$

But $\zeta\left( \tau_{c} \right)=0$

$$\Longrightarrow3{\sigma_{c}}^{2}-a_{2}-2h_{1}\varphi_{c}\sin\varphi_{c}\tau_{c}- h_{2}\cos\varphi_{c}\tau_{c}=\mathcal{B}_{1}$$

Also from equation (35) we have $e^{\varphi}=e^{0}=1$. Selecting $\varphi$ from (35) and differentiating with respect to $\tau$ and evaluating at $\tau=\tau_{c}$ for which $\zeta\left( \tau_{c} \right)=0$ and $\varphi\left( \tau_{c} \right)=\sigma_{c}$ we get

$$h_{1}\varphi^{2}\sin\varphi\tau-h_{3}\sin\varphi\tau+h_{2}\varphi\cos\varphi\tau=\varphi^{3}-a_{2}\varphi(38)$$

Then,

$$\frac{d\varphi}{d\tau}|_{\tau{=\tau}_{c}}=2h_{1}\varphi_{c}\sin\varphi_{c}\tau_{c}+h_{1}\tau_{c}{\varphi_{c}}^{2}\cos\varphi_{c}\tau_{c}-\tau_{c}h_{3}\cos\varphi_{c}\tau_{c}-h_{2}\tau_{c}\varphi_{c}\sin\varphi_{c}\tau_{c}+h_{2}\cos\varphi_{c}\tau_{c}=3{\varphi_{c}}^{2}-a_{2}$$

$$\Longrightarrow a_{2}-3{\varphi_{c}}^{2}+\left( h_{2}+h_{1}\tau_{c}{\varphi_{c}}^{2}-\tau_{c}h_{3} \right)\cos\varphi_{c}\tau_{c}+\left( 2h_{1}\varphi_{c}-h_{2}\tau_{c}\varphi_{c} \right)\sin\varphi_{c}\tau_{c}=\mathcal{B}_{2}$$

Again, selecting $\zeta$ from (30), then and differentiating with respect to $\tau$ and evaluating at $\tau=\tau_{c}$ for which $\zeta\left( \tau_{c} \right)=0$ and $\varphi\left( \tau_{c} \right)=\varphi_{c}$ we have

$$-h_{2}\zeta\cos\varphi\tau-h_{1}\zeta^{2}\sin\varphi\tau+2h_{1}\varphi\tau\cos\tau\varphi=-2a_{1}\zeta\varphi-3\zeta^{2}\varphi(39)$$

Then,

$$\frac{d\zeta}{d\tau}|_{\tau{=\tau}_{c}}=-h_{2}\sin\varphi_{c}\tau_{c}-h_{2}\tau_{c}\zeta_{c}\cos\varphi_{c}\tau_{c}-2h_{1}\zeta_{c}\sin\varphi_{c}\tau_{c}-h_{1}\tau_{c}{\zeta_{c}}^{2}\sin\varphi_{c}\tau_{c}$$

$$+2h_{1}\varphi_{c}\cos\varphi_{c}\tau_{c}-2h_{1}\zeta_{c}\varphi_{c}\sin\varphi_{c}\tau_{c}=-2a_{1}\varphi_{c}-6\zeta_{c}\varphi_{c}$$

$$\Longrightarrow-h_{2}\sin\varphi_{c}\tau_{c}+2h_{1}\varphi_{c}\cos\varphi_{c}\tau_{c}=-2a_{1}\varphi_{c} \Longrightarrow2a_{1}\varphi_{c}-h_{2}\sin\varphi_{c}\tau_{c}+2h_{1}\varphi_{c}\cos\varphi_{c}\tau_{c}=\mathcal{B}_{2}$$

At this point, we solve for the difference of $\mathcal{B}_{1} and \mathcal{B}_{2}$ and the sum of $\mathcal{B}_{2} and \mathcal{B}_{1}$, that is
$\mathcal{B}_{1}\frac{d\varphi}{d\tau}|_{\tau{=\tau}_{c}}-\mathcal{B}_{2}\frac{d\zeta}{d\tau}|_{\tau{=\tau}_{c}}$ and $\mathcal{B}_{2}\frac{d\varphi}{d\tau}|_{\tau{=\tau}_{c}}+\mathcal{B}_{1}\frac{d\zeta}{d\tau}|_{\tau{=\tau}_{c}}$ respectively, then we obtain

$$\mathcal{B}_{1}\frac{d\zeta}{d\tau}|_{\tau{=\tau}_{c}}-\mathcal{B}_{2}\frac{d\zeta}{d\tau}|_{\tau{=\tau}_{c}}$$

$$\Longrightarrow\left[ 2a_{1}\varphi_{c}+\left( 2h_{1}\varphi_{c}-\tau_{c}h_{2}\varphi_{c} \right)\cos\varphi_{c}\tau_{c}+\left( h_{3}\tau_{c}-\tau_{c}h_{1}{\varphi_{c}}^{2}-h_{2} \right)\sin\varphi_{c}\tau_{c} \right]-\left[ 2a_{1}\varphi_{c}-h_{2}\sin\varphi_{c}\tau_{c}+2h_{1}\varphi_{c}\cos\varphi_{c}\tau_{c} \right]$$

$$\Longrightarrow-\tau_{c}h_{2}\varphi_{c}\cos\varphi_{c}\tau_{c}+\left( h_{3}\tau_{c}-\tau_{c}h_{1}{\varphi_{c}}^{2} \right)\sin\varphi_{c}\tau_{c}$$

Since$\tau_{c}=\varphi_{c}$, we have

$$\Longrightarrow h_{2}{\varphi_{c}}^{2}\cos\varphi_{c}\tau_{c}+\left( h_{1}{\varphi_{c}}^{3}-h_{3}\varphi_{c} \right)\sin\varphi_{c}\tau_{c}$$

Therefore,

$$\mathcal{B}_{3}=h_{2}{\varphi_{c}}^{2} and \mathcal{B}_{4}=h_{1}{\varphi_{c}}^{3}-h_{3}\varphi_{c}$$

Also

$$\mathcal{B}_{2}\frac{d\varphi}{d\tau}|_{\tau{=\tau}_{c}}+\mathcal{B}_{1}\frac{d\zeta}{d\tau}|_{\tau{=\tau}_{c}}$$

$$\Longrightarrow\left[ d_{2}-3{\varphi_{c}}^{2}+\left( h_{2}+h_{1}\tau_{c}{\varphi_{c}}^{2}-\tau_{c}h_{3} \right)\cos\varphi_{c}\tau_{c}+\left( 2h_{1}\varphi_{c}-h_{2}\tau_{c}\sigma_{c} \right)\sin\varphi_{c}\tau_{c} \right]$$

$$+\left[ 3{\varphi_{c}}^{2}-a_{2}-2h_{1}\varphi\sin\varphi_{c}\tau_{c}- h_{2}\cos\varphi_{c}\tau_{c} \right]$$

Again remember that $\tau_{c}=\varphi_{c},$ and we then obtain

$$-h_{2}{\varphi_{c}}^{2}\sin\varphi_{c}\tau_{c}+\left( h_{1}{\varphi_{c}}^{3}-h_{3}\varphi_{c} \right)\cos\varphi_{c}\tau_{c} \Longrightarrow h_{2}{\varphi_{c}}^{2}\sin\varphi_{c}\tau_{c}-\left( h_{1}{\varphi_{c}}^{3}-h_{3}\varphi_{c} \right)\cos\varphi_{c}\tau_{c}$$

Therefore,

$$\mathcal{B}_{3}=h_{2}{\varphi_{c}}^{2} and-\mathcal{B}_{4}=\left( h_{1}{\varphi_{c}}^{3}-h_{3}\varphi_{c} \right)$$

The result of the differentiations, the difference and sum when put together, yields

$$\mathcal{B}_{2}\frac{d\varphi}{d\tau}|_{\tau{=\tau}_{c}}+\mathcal{B}_{1}\frac{d\zeta}{d\tau}|_{\tau{=\tau}_{c}}=\mathcal{B}_{3}\cos\varphi_{c}\tau_{c}+\mathcal{B}_{4}\sin\varphi_{c}\tau_{c} (40)$$

$$\mathcal{B}_{2}\frac{d\varphi}{d\tau_{1}}|_{\tau{=\tau}_{c}}+\mathcal{B}_{1}\frac{d\zeta}{d\tau}|_{\tau{=\tau}_{c}}=\mathcal{B}_{3}\sin\varphi_{c}\tau_{c}-\mathcal{B}_{4}\cos\varphi_{c}\tau_{c} (41)$$

To solve equations (40) and (41) simultaneously, we multiply equation (40) by $\mathcal{B}_{2}$ and equation (41) by $\mathcal{B}_{1}$, then subtract the product of (40) from (41) and this eliminates $\frac{d\varphi}{d\tau}$ .Therefore, we have

$$\mathcal{B}_{1}\mathcal{B}_{2}\frac{d\varphi}{d\tau}|_{\tau{=\tau}_{c}}+\mathcal{B}_{1}^{2}\frac{d\zeta}{d\tau}|_{\tau{=\tau}_{c}}=\mathcal{B}_{1}\mathcal{B}_{3}\sin\varphi_{c}\tau_{c}-\mathcal{B}_{1}\mathcal{B}_{4}\cos\varphi_{c}\tau_{c}$$

$$\mathcal{B}_{1}\mathcal{B}_{2}\frac{d\varphi}{d\tau}|_{\tau{=\tau}_{c}}-\mathcal{B}_{2}^{2}\frac{d\zeta}{d\tau}|_{\tau{=\tau}_{c}}=\mathcal{B}_{2}\mathcal{B}_{3}\cos\varphi_{c}\tau_{c}+\mathcal{B}_{2}\mathcal{B}_{4}\sin\varphi_{c}\tau_{c}$$

$$\Longrightarrow\left( \mathcal{B}_{1}^{2}+\mathcal{B}_{2}^{2} \right)\frac{d\zeta}{d\tau}|_{\tau{=\tau}_{c}}=\left( \mathcal{B}_{1}\mathcal{B}_{3}-\mathcal{B}_{2}\mathcal{B}_{4} \right)\sin\varphi_{c}\tau_{c}-(\mathcal{B}_{1}\mathcal{B}_{4}+\mathcal{B}_{2}\mathcal{B}_{3})\cos\varphi_{c}\tau_{c}$$

$$\frac{d\zeta}{d\tau}|_{\tau{=\tau}_{c}}=\frac{\left( \mathcal{B}_{1}\mathcal{B}_{3}-\mathcal{B}_{2}\mathcal{B}_{4} \right)\sin\sigma_{c}\tau_{c}-(\mathcal{B}_{1}\mathcal{B}_{4}+\mathcal{B}_{2}\mathcal{B}_{3})\cos\varphi_{c}\tau_{c}}{\mathcal{B}_{1}^{2}+\mathcal{B}_{2}^{2}} (42)$$

Let us solve equations (26) simultaneously.

$$h_{2}\varphi_{c}\sin\varphi_{c}\tau_{c}+\left( h_{3}-h_{1}\varphi_{c}^{2} \right)\cos\varphi_{c}\tau_{c}=a_{1}\varphi_{c}^{2}-a_{3}$$

$$h_{2}\varphi_{c}\cos\varphi_{c}\tau_{c}-\left( h_{3}-h_{1}\varphi_{c}^{2} \right)\sin\varphi_{c}\tau_{c}=\varphi_{c}^{3}-a_{2}\varphi_{c}$$

If we multiply the first sub equation of (26) by $h_{3}-h_{1}\varphi_{c}^{2}$ and the second sub equation of (26) by $h_{2}\varphi_{c}$ and add the resulting products, we obtain

$$h_{2}\varphi_{c}\left( h_{3}-h_{1}\varphi_{c}^{2} \right)\sin\varphi_{c}\tau_{c}+\left( h_{3}-h_{1}\varphi_{c}^{2} \right)^{2}\cos\varphi_{c}\tau_{c}=\left( a_{1}\varphi_{c}^{2}-a_{3} \right)\left( h_{3}-h_{1}\varphi_{c}^{2} \right)$$

$$h_{2}^{2}\varphi_{c}^{2}\cos\varphi_{c}\tau_{c}-h_{2}\varphi_{c}\left( h_{3}-h_{1}\varphi_{c}^{2} \right)\sin\varphi_{c}\tau_{c}=h_{2}\varphi_{c}\left( \varphi_{c}^{3}-a_{2}\varphi_{c} \right)$$

$$\Longrightarrow h_{2}^{2}\varphi_{c}^{2}\cos\varphi_{c}\tau_{c}+\left( h_{3}-h_{1}\varphi_{c}^{2} \right)^{2}\cos\varphi_{c}\tau_{c}=\left( a_{1}\varphi_{c}^{2}-a_{3} \right)\left( h_{3}-h_{1}\varphi_{c}^{2} \right)+h_{2}\varphi_{c}\left( \varphi_{c}^{3}-a_{2}\varphi_{c} \right)$$

$$\left[ h_{2}^{2}\varphi_{c}^{2}+\left( h_{3}-h_{1}\varphi_{c}^{2} \right)^{2} \right]\cos\varphi_{c}\tau_{c}=\left( a_{1}\varphi_{c}^{2}-a_{3} \right)\left( h_{3}-h_{1}\varphi_{c}^{2} \right)+h_{2}\varphi_{c}\left( \varphi_{c}^{3}-a_{2}\varphi_{c} \right)$$

$$\Longrightarrow\cos\varphi_{c}\tau_{c}=\frac{h_{2}\varphi_{c}\left( \varphi_{c}^{3}-a_{2}\varphi_{c} \right)+\left( a_{1}\varphi_{c}^{2}-a_{3} \right)\left( h_{3}-h_{1}\varphi_{c}^{2} \right)}{\left[ h_{2}^{2}\varphi_{c}^{2}+\left( h_{3}-h_{1}\varphi_{c}^{2} \right)^{2} \right]} (43)$$

On the other hand, if we multiply the first sub equation of (26) by $h_{2}\varphi_{c}$ and the second sub equation of (26) by $h_{3}-h_{1}\varphi_{c}^{2}$ and also add the resulting product, we obtain

$$h_{2}^{2}\varphi_{c}^{2}\sin\varphi_{c}\tau_{c}+h_{2}\varphi_{c}\left( h_{3}-h_{1}\varphi_{c}^{2} \right)\cos\varphi_{c}\tau_{c}=h_{2}\varphi_{c}\left( a_{1}\varphi_{c}^{2}-a_{3} \right)$$

$$-h_{2}\varphi_{c}\left( h_{3}-h_{1}\varphi_{c}^{2} \right)\cos\varphi_{c}\tau_{c}+\left( h_{3}-h_{1}\varphi_{c}^{2} \right)^{2}\sin\varphi_{c}\tau_{c}=-\left( h_{3}-h_{1}\varphi_{c}^{2} \right)\left( \varphi_{c}^{3}-a_{2}\varphi_{c} \right)$$

$$\Longrightarrow\left[ h_{2}^{2}\varphi_{c}^{2}+\left( h_{3}-h_{1}\varphi_{c}^{2} \right)^{2} \right]\sin\varphi_{c}\tau_{c}=h_{2}\varphi_{c}\left( a_{1}\varphi_{c}^{2}-a_{3} \right)-\left( h_{3}-h_{1}\varphi_{c}^{2} \right)\left( \varphi_{c}^{3}-a_{2}\varphi_{c} \right)$$

$$\sin\sigma_{c}\tau_{c}=\frac{h_{2}\varphi_{c}\left( a_{1}\varphi_{c}^{2}-a_{3} \right)-\left( h_{3}-h_{1}\varphi_{c}^{2} \right)\left( \varphi_{c}^{3}-a_{2}\varphi_{c} \right)}{\left[ h_{2}^{2}\varphi_{c}^{2}+\left( h_{3}-h_{1}\varphi_{c}^{2} \right)^{2} \right]} (44)$$

Substituting (43) and (44) into equation (42) it yields (45) below as shown below

$$Let 2a_{1}{\sigma\varphi}_{c}+\left( 2h_{1}\varphi_{c}-\tau_{c}h_{2}\varphi_{c} \right)\cos\varphi_{c}\tau_{c}+\left( h_{3}\tau_{c}-\tau_{c}h_{1}{\varphi_{c}}^{2}-h_{2} \right)\sin\varphi_{c}\tau_{c}=\mathcal{B}_{1}$$

$$a_{2}-3{\varphi_{c}}^{2}+\left( h_{2}+h_{1}\tau_{c}{\varphi_{c}}^{2}-\tau_{c}h_{3} \right)\cos\varphi_{c}\tau_{c}+\left( 2h_{1}\varphi_{c}-h_{2}\tau_{c}\varphi_{c} \right)\sin\varphi_{c}\tau_{c}$$

$$h_{2}{\varphi_{c}}^{2}=\mathcal{B}_{3} and h_{1}{\varphi_{c}}^{3}-h_{3}\varphi_{c}=\mathcal{B}_{4}$$

Therefore,

$$\frac{\begin{aligned} \left[ \begin{aligned} \left[ 2a_{1}\varphi_{c}+\left( 2h_{1}\varphi_{c}-\tau_{c}h_{2}\varphi_{c} \right)\cos\varphi_{c}\tau_{c}+\left( h_{3}\tau_{c}-\tau_{c}h_{1}{\varphi_{c}}^{2}-h_{2} \right)\sin\varphi_{c}\tau_{c} \right]\left[ h_{2}{\varphi_{c}}^{2} \right] \\ -\left[ a_{2}-3{\varphi_{c}}^{2}+\left( h_{2}+h_{1}\tau_{c}{\varphi_{c}}^{2}-\tau_{c}h_{3} \right)\cos\varphi_{c}\tau_{c}+\left( 2h_{1}\varphi_{c}-h_{2}\tau_{c}\varphi_{c} \right)\sin\varphi_{c}\tau_{c} \right]\left[ h_{1}{\varphi_{c}}^{3}-h_{3}\varphi_{c} \right] \end{aligned} \right]\left[ \sin\varphi_{c}\tau_{c} \right] \\ -\left[ \begin{aligned} \left[ 2a_{1}\varphi_{c}+\left( 2h_{1}\varphi_{c}-\tau_{c}h_{2}\varphi_{c} \right)\cos\varphi_{c}\tau_{c}+\left( h_{3}\tau_{c}-\tau_{c}h_{1}{\varphi_{c}}^{2}-h_{2} \right)\sin\varphi_{c}\tau_{c} \right]\left[ h_{1}{\varphi_{c}}^{3}-h_{3}\varphi_{c} \right] \\ +\left[ a_{2}-3{\varphi_{c}}^{2}+\left( h_{2}+h_{1}\tau_{c}{\varphi_{c}}^{2}-\tau_{c}h_{3} \right)\cos\varphi_{c}\tau_{c}+\left( 2h_{1}\varphi_{c}-h_{2}\tau_{c}\varphi_{c} \right)\sin\varphi_{c}\tau_{c} \right]\left[ h_{2}{\varphi_{c}}^{2} \right] \end{aligned} \right][\cos\varphi_{c}\tau_{c}] \end{aligned}}{\mathcal{B}_{1}^{2}+\mathcal{B}_{2}^{2}}$$

$$\Longrightarrow$$

$$\frac{\begin{aligned} \left[ \begin{aligned} 2a_{1}{h_{2}\varphi_{c}}^{3}+2h_{1}h_{2}{\varphi_{c}}^{3}\cos\varphi_{c}\tau_{c}-\tau_{c}h_{2}^{2}{\varphi_{c}}^{3}\cos\varphi_{c}\tau_{c}+\tau_{c}h_{2}h_{3}{\varphi_{c}}^{2}\sin\varphi_{c}\tau_{c} \\ -\tau_{c}h_{1}h_{2}{\varphi_{c}}^{4}\sin\varphi_{c}\tau_{c}{-h}_{2}^{2}{\varphi_{c}}^{2}\sin\varphi_{c}\tau_{c}-a_{2}h_{1}{\varphi_{c}}^{3}+3h_{1}{\varphi_{c}}^{5}-h_{1}h_{2}{\varphi_{c}}^{3}\cos\varphi_{c}\tau_{c} \\ -\tau_{c}h_{1}^{2}{\varphi_{c}}^{5}\cos\varphi_{c}\tau_{c}+\tau_{c}h_{1}h_{3}{\varphi_{c}}^{3}\cos\varphi_{c}\tau_{c}-2h_{1}^{2}{\varphi_{c}}^{4}\sin\varphi_{c}\tau_{c}+\tau_{c}h_{1}h_{2}{\varphi_{c}}^{4}\sin\varphi_{c}\tau_{c} \\ +a_{2}h_{3}\varphi_{c}-3h_{3}{\varphi_{c}}^{3}+h_{2}h_{3}\varphi_{c}\cos\varphi_{c}\tau_{c}+\tau_{c}h_{1}h_{3}{\varphi_{c}}^{3}\cos\varphi_{c}\tau_{c} \\ -\tau_{c}h_{3}^{2}\varphi_{c}\cos\varphi_{c}\tau_{c}+2h_{1}h_{3}{\varphi_{c}}^{2}\sin\varphi_{c}\tau_{c}-\tau_{c}h_{2}h_{3}{\varphi_{c}}^{2}\sin\varphi_{c}\tau_{c} \end{aligned} \right]\left[ \sin\varphi_{c}\tau_{c} \right] \\ +\left[ \begin{aligned} -2a_{1}{h_{1}\varphi_{c}}^{4}-2h_{1}^{2}{\varphi_{c}}^{4}\cos\varphi_{c}\tau_{c}+\tau_{c}h_{1}h_{2}{\varphi_{c}}^{4}\cos\varphi_{c}\tau_{c}-\tau_{c}h_{1}h_{3}{\varphi_{c}}^{3}\sin\varphi_{c}\tau_{c} \\ +h_{1}h_{2}{\varphi_{c}}^{3}\sin\varphi_{c}\tau_{c}+\tau_{c}h_{1}^{2}{\varphi_{c}}^{5}\sin\varphi_{c}\tau_{c}+2a_{1}{h_{3}\varphi_{c}}^{2}+2h_{1}h_{3}{\varphi_{c}}^{2}\cos\varphi_{c}\tau_{c} \\ -\tau_{c}h_{2}h_{3}{\varphi_{c}}^{2}\cos\varphi_{c}\tau_{c}+\tau_{c}h_{3}^{2}\varphi_{c}\sin\varphi_{c}\tau_{c}-h_{2}h_{3}\varphi_{c}\sin\varphi_{c}\tau_{c} \\ -\tau_{c}h_{1}h_{3}{\varphi_{c}}^{3}\sin\varphi_{c}\tau_{c}-a_{2}h_{2}{\varphi_{c}}^{2}+3h_{2}{\varphi_{c}}^{4}-h_{2}^{2}{\varphi_{c}}^{2}\cos\varphi_{c}\tau_{c}-\tau_{c}h_{1}h_{2}{\varphi_{c}}^{4}\cos\varphi_{c}\tau_{c} \\ +\tau_{c}h_{2}h_{3}{\varphi_{c}}^{2}\cos\varphi_{c}\tau_{c}-2h_{1}h_{2}{\varphi_{c}}^{3}\sin\varphi_{c}\tau_{c}+\tau_{c}h_{2}^{2}{\varphi_{c}}^{2}\sin\varphi_{c}\tau_{c} \end{aligned} \right]\left[ \cos\varphi_{c}\tau_{c} \right] \end{aligned}}{\mathcal{B}_{1}^{2}+\mathcal{B}_{2}^{2}}$$

$$\Longrightarrow$$

$$\frac{\begin{aligned} \begin{aligned} 2a_{1}{h_{2}\varphi_{c}}^{3}\sin\varphi_{c}\tau_{c}+2h_{1}h_{2}{\varphi_{c}}^{3}\cos\varphi_{c}\tau_{c}\sin\varphi_{c}\tau_{c}-\tau_{c}h_{2}^{2}{\varphi_{c}}^{3}\cos\varphi_{c}\tau_{c}\sin\varphi_{c}\tau_{c}+\tau_{c}h_{2}h_{3}{\varphi_{c}}^{2}{sin}^{2} \varphi_{c}\tau_{c} \\ -\tau_{c}h_{1}h_{2}{\varphi_{c}}^{4}{sin}^{2} \varphi_{c}\tau_{c}{-h}_{2}^{2}{\varphi_{c}}^{2}{sin}^{2} \varphi_{c}\tau_{c}-a_{2}h_{1}{\varphi_{c}}^{3}\sin\varphi_{c}\tau_{c}+3h_{1}{\varphi_{c}}^{5}\sin\varphi_{c}\tau_{c}-h_{1}h_{2}{\varphi_{c}}^{3}\cos\varphi_{c}\tau_{c}\sin\varphi_{c}\tau_{c} \\ -\tau_{c}h_{1}^{2}{\varphi_{c}}^{5}\cos\varphi_{c}\tau_{c}\sin\varphi_{c}\tau_{c}+\tau_{c}h_{1}h_{3}{\varphi_{c}}^{3}\cos\varphi_{c}\tau_{c}\sin\varphi_{c}\tau_{c}-2h_{1}^{2}{\varphi_{c}}^{4}{sin}^{2} \varphi_{c}\tau_{c}+\tau_{c}h_{1}h_{2}{\varphi_{c}}^{4}{sin}^{2} \varphi_{c}\tau_{c} \\ +a_{2}h_{3}\varphi_{c}\sin\varphi_{c}\tau_{c}-3h_{3}{\varphi_{c}}^{3}\sin\varphi_{c}\tau_{c}+h_{2}h_{3}\varphi_{c}\cos\varphi_{c}\tau_{c}\sin\varphi_{c}\tau_{c}+\tau_{c}h_{1}h_{3}{\varphi_{c}}^{3}\cos\varphi_{c}\tau_{c}\sin\varphi_{c}\tau_{c} \\ -\tau_{c}h_{3}^{2}\varphi_{c}\cos\varphi_{c}\tau_{c}\sin\varphi_{c}\tau_{c}+2h_{1}h_{3}{\varphi_{c}}^{2}{sin}^{2} \varphi_{c}\tau_{c}-\tau_{c}h_{2}h_{3}{\varphi_{c}}^{2}{sin}^{2} \varphi_{c}\tau_{c} \end{aligned} \\ \begin{aligned} -2a_{1}{h_{1}\varphi_{c}}^{4}\cos\varphi_{c}\tau_{c}-2h_{1}^{2}{\varphi_{c}}^{4}{cos}^{2} \varphi_{c}\tau_{c}+\tau_{c}h_{1}h_{2}{\varphi_{c}}^{4}{cos}^{2} \varphi_{c}\tau_{c}-\tau_{c}h_{1}h_{3}{\varphi_{c}}^{3}\cos\varphi_{c}\tau_{c}\sin\varphi_{c}\tau_{c} \\ +h_{1}h_{2}{\varphi_{c}}^{3}\cos\varphi_{c}\tau_{c}\sin\varphi_{c}\tau_{c}+\tau_{c}h_{1}^{2}{\varphi_{c}}^{5}\cos\varphi_{c}\tau_{c}\sin\varphi_{c}\tau_{c}+2a_{1}{h_{3}\varphi_{c}}^{2}\cos\varphi_{c}\tau_{c}+2h_{1}h_{3}{\varphi_{c}}^{2}{cos}^{2} \varphi_{c}\tau_{c} \\ -\tau_{c}h_{2}h_{3}{\varphi_{c}}^{2}{cos}^{2} \varphi_{c}\tau_{c}+\tau_{c}h_{3}^{2}\varphi_{c}\cos\varphi_{c}\tau_{c}\sin\varphi_{c}\tau_{c}-h_{2}h_{3}\varphi_{c}\cos\varphi_{c}\tau_{c}\sin\varphi_{c}\tau_{c} \\ -\tau_{c}h_{1}h_{3}{\varphi_{c}}^{3}\cos\varphi_{c}\tau_{c}\sin\varphi_{c}\tau_{c}-a_{2}h_{2}{\varphi_{c}}^{2}\cos\varphi_{c}\tau_{c}+3h_{2}{\varphi_{c}}^{4}\cos\varphi_{c}\tau_{c}-h_{2}^{2}{\varphi_{c}}^{2}{cos}^{2} \varphi_{c}\tau_{c}-\tau_{c}h_{1}h_{2}{\varphi_{c}}^{4}{cos}^{2} \varphi_{c}\tau_{c} \\ +\tau_{c}h_{2}h_{3}{\varphi_{c}}^{2}{cos}^{2} \varphi_{c}\tau_{c}-2h_{1}h_{2}{\varphi_{c}}^{3}\cos\varphi_{c}\tau_{c}\sin\varphi_{c}\tau_{c}+\tau_{c}h_{2}^{2}{\varphi_{c}}^{2}\cos\varphi_{c}\tau_{c}\sin\varphi_{c}\tau_{c} \end{aligned} \end{aligned}}{\mathcal{B}_{1}^{2}+\mathcal{B}_{2}^{2}}$$

$$\frac{\begin{aligned} \Longrightarrow\left[ 3h_{1}\sin\varphi_{c}\tau_{c}-\tau_{c}h_{1}^{2}\cos\varphi_{c}\tau_{c}\sin\varphi_{c}\tau_{c}+\tau_{c}h_{1}^{2}\cos\varphi_{c}\tau_{c}\sin\varphi_{c}\tau_{c} \right]{\varphi_{c}}^{5} \\ +\left[ \begin{aligned} -\tau_{c}h_{1}h_{2}{sin}^{2}\varphi_{c}\tau_{c}-2h_{1}^{2}{sin}^{2}\varphi_{c}\tau_{c}+\tau_{c}h_{1}h_{2}{sin}^{2}\varphi_{c}\tau_{c}-2a_{1}h_{1}\cos\varphi_{c}\tau_{c}+3h_{2}\cos\varphi_{c}\tau_{c} \\ -\tau_{c}h_{1}h_{2}{\varphi_{c}}^{4}{cos}^{2}\varphi_{c}\tau_{c}-2h_{1}^{2}{cos}^{2}\varphi_{c}\tau_{c}+\tau_{c}h_{1}h_{2}{cos}^{2}\varphi_{c}\tau_{c} \end{aligned} \right]{\varphi_{c}}^{4} \\ +\left[ \begin{aligned} 2a_{1}h_{2}\sin\varphi_{c}\tau_{c}+2h_{1}h_{2}\cos\varphi_{c}\tau_{c}\sin\varphi_{c}\tau_{c}-\tau_{c}h_{2}^{2}\cos\varphi_{c}\tau_{c}\sin\varphi_{c}\tau_{c}-a_{2}h_{1}\sin\varphi_{c}\tau_{c} \\ -h_{1}h_{2}\cos\varphi_{c}\tau_{c}\sin\varphi_{c}\tau_{c}+\tau_{c}h_{1}h_{3}\cos\varphi_{c}\tau_{c}\sin\varphi_{c}\tau_{c}-3h_{3}\sin\varphi_{c}\tau_{c} \\ +\tau_{c}h_{1}h_{3}\cos\varphi_{c}\tau_{c}\sin\varphi_{c}\tau_{c}-\tau_{c}h_{1}h_{3}\cos\varphi_{c}\tau_{c}\sin\varphi_{c}\tau_{c}+h_{1}h_{2}\cos\varphi_{c}\tau_{c}\sin\varphi_{c}\tau_{c} \\ -\tau_{c}h_{1}h_{3}{\varphi_{c}}^{3}\cos\varphi_{c}\tau_{c}\sin\varphi_{c}\tau_{c}-2h_{1}h_{2}\cos\varphi_{c}\tau_{c}\sin\varphi_{c}\tau_{c}+\tau_{c}h_{2}^{2}\cos\varphi_{c}\tau_{c}\sin\varphi_{c}\tau_{c} \end{aligned} \right]{\varphi_{c}}^{3} \\ +\left[ \begin{aligned} \tau_{c}h_{2}h_{3}{sin}^{2}\varphi_{c}\tau_{c}{-h}_{2}^{2}{sin}^{2}\varphi_{c}\tau_{c}+2h_{1}h_{3}{sin}^{2}\varphi_{c}\tau_{c}-\tau_{c}h_{2}h_{3}{sin}^{2}\varphi_{c}\tau_{c} \\ +2a_{1}h_{3}\cos\varphi_{c}\tau_{c}+2h_{1}h_{3}{cos}^{2}\varphi_{c}\tau_{c}-\tau_{c}h_{2}h_{3}{cos}^{2}\varphi_{c}\tau_{c}-a_{2}h_{2}\cos\varphi_{c}\tau_{c} \\ -h_{2}^{2}{cos}^{2}\varphi_{c}\tau_{c}+\tau_{c}h_{2}h_{3}{cos}^{2}\varphi_{c}\tau_{c} \end{aligned} \right]{\varphi_{c}}^{2} \\ \left[ \begin{aligned} a_{2}h_{3}\sin\varphi_{c}\tau_{c}+h_{2}h_{3}\cos\varphi_{c}\tau_{c}\sin\varphi_{c}\tau_{c}-\tau_{c}h_{3}^{2}\cos\varphi_{c}\tau_{c}\sin\varphi_{c}\tau_{c} \\ +\tau_{c}h_{3}^{2}\cos\varphi_{c}\tau_{c}\sin\varphi_{c}\tau_{c}-h_{2}h_{3}\cos\varphi_{c}\tau_{c}\sin\varphi_{c}\tau_{c} \end{aligned} \right]\varphi_{c} \end{aligned}}{\mathcal{B}_{1}^{2}+\mathcal{B}_{2}^{2}}$$

$$\frac{\begin{aligned} \Longrightarrow\left[ 3h_{1}\sin\varphi_{c}\tau_{c} \right]{\varphi_{c}}^{5} \\ +\left[ 3h_{2}\cos\varphi_{c}\tau_{c}-2a_{1}h_{1}\cos\varphi_{c}\tau_{c}-2h_{1}^{2}\left[ {sin}^{2}\varphi_{c}\tau_{c}+{cos}^{2}\varphi_{c}\tau_{c} \right] \right]{\varphi_{c}}^{4} \\ +\left[ 2a_{1}h_{2}\sin\varphi_{c}\tau_{c}-a_{2}h_{1}\sin\varphi_{c}\tau_{c}-3h_{3}\sin\varphi_{c}\tau_{c} \right]{\varphi_{c}}^{3} \\ +\left[ 2a_{1}h_{3}\cos\varphi_{c}\tau_{c}-a_{2}h_{2}\cos\varphi_{c}\tau_{c}+\left[ 2h_{1}h_{3}-h_{2}^{2} \right]\left[ {sin}^{2}\varphi_{c}\tau_{c}+{cos}^{2}\varphi_{c}\tau_{c} \right] \right]{\varphi_{c}}^{2} \\ +\left[ a_{2}h_{3}\sin\varphi_{c}\tau_{c} \right]\varphi_{c} \end{aligned}}{\mathcal{B}_{1}^{2}+\mathcal{B}_{2}^{2}}$$

But ${sin}^{2}\sigma_{c}\tau_{c}+{cos}^{2}\sigma_{c}\tau_{c}=1,$ therefore the above changes to

$$\frac{\begin{aligned} \Longrightarrow\left[ 3h_{1}\sin\varphi_{c}\tau_{c} \right]{\varphi_{c}}^{5} \\ +\left[ 3h_{2}\cos\varphi_{c}\tau_{c}-2a_{1}h_{1}\cos\varphi_{c}\tau_{c}-2h_{1}^{2} \right]{\varphi_{c}}^{4} \\ +\left[ 2a_{1}h_{2}\sin\varphi_{c}\tau_{c}-a_{2}h_{1}\sin\varphi_{c}\tau_{c}-3h_{3}\sin\varphi_{c}\tau_{c} \right]{\varphi_{c}}^{3} \\ +\left[ 2a_{1}h_{3}\cos\varphi_{c}\tau_{c}-a_{2}h_{2}\cos\varphi_{c}\tau_{c}+\left[ 2h_{1}h_{3}-h_{2}^{2} \right] \right]{\varphi_{c}}^{2} \\ +\left[ a_{2}h_{3}\sin\varphi_{c}\tau_{c} \right]\varphi_{c} \end{aligned}}{\mathcal{B}_{1}^{2}+\mathcal{B}_{2}^{2}}$$

Now, equations (35) and (36) will be substituted into the above equation to obtain

$$\frac{3k_{1}{\varphi_{c}}^{5}\left[ a_{1}h_{2}\varphi_{c}^{3}-a_{3}h_{2}\varphi_{c}-h_{3}\varphi_{c}^{3}+a_{2}h_{3}\varphi_{c}+h_{1}\varphi_{c}^{5}-a_{2}h_{1}\varphi_{c}^{3} \right]}{\left[ h_{2}^{2}\varphi_{c}^{2}+h_{3}^{2}-2h_{1}h_{3}\varphi_{c}^{2}+h_{1}^{2}\varphi_{c}^{4} \right]\left[ \mathcal{B}_{1}^{2}+\mathcal{B}_{2}^{2} \right]}$$

$$+\frac{\begin{aligned} \left[ 3h_{2}{\varphi_{c}}^{4}-2a_{1}h_{1}{\varphi_{c}}^{4} \right]\left[ a_{1}h_{3}\varphi_{c}^{2}-a_{1}h_{1}\varphi_{c}^{4}-a_{3}h_{3}+a_{3}h_{1}\varphi_{c}^{2}+h_{2}\varphi_{c}^{4}-a_{2}h_{2}\varphi_{c}^{2} \right] \\ -2h_{1}^{2}{\varphi_{c}}^{4}\left[ h_{2}^{2}\varphi_{c}^{2}+h_{3}^{2}-2h_{1}h_{3}\varphi_{c}^{2}+h_{1}^{2}\varphi_{c}^{4} \right] \end{aligned}}{\left[ h_{2}^{2}\varphi_{c}^{2}+h_{3}^{2}-2h_{1}h_{3}\varphi_{c}^{2}+h\varphi_{c}^{4} \right]\left[ \mathcal{B}_{1}^{2}+\mathcal{B}_{2}^{2} \right]}$$

$$+\frac{\left[ 2a_{1}h_{2}{\varphi_{c}}^{3}-a_{2}h_{1}{\varphi_{c}}^{3}-3h_{3}{\varphi_{c}}^{3} \right]\left[ a_{1}h_{2}\varphi_{c}^{3}-a_{3}h_{2}\varphi_{c}-h_{3}\varphi_{c}^{3}+a_{2}h_{3}\varphi_{c}+h_{1}\varphi_{c}^{5}-a_{2}h_{1}\varphi_{c}^{3} \right]}{\left[ h_{2}^{2}\varphi_{c}^{2}+h_{3}^{2}-2h_{1}h_{3}\varphi_{c}^{2}+h_{1}^{2}\varphi_{c}^{4} \right]\left[ \mathcal{B}_{1}^{2}+\mathcal{B}_{2}^{2} \right]}$$

$$+\frac{\begin{aligned} \left[ 2a_{1}h_{3}{\varphi_{c}}^{2}-a_{2}h_{2}{\varphi_{c}}^{2} \right]\left[ a_{1}h_{3}\varphi_{c}^{2}-a_{1}h_{1}\varphi_{c}^{4}-a_{3}h_{3}+a_{3}h_{1}\varphi_{c}^{2}+h_{2}\varphi_{c}^{4}-a_{2}h_{2}\varphi_{c}^{2} \right] \\ +\left[ 2h_{1}h_{3}{\varphi_{c}}^{2}-h_{2}^{2}{\varphi_{c}}^{2} \right]\left[ h_{2}^{2}\varphi_{c}^{2}+h_{3}^{2}-2h_{1}h_{3}\varphi_{c}^{2}+h_{1}^{2}\varphi_{c}^{4} \right] \end{aligned}}{\left[ h_{2}^{2}\varphi_{c}^{2}+h_{3}^{2}-2h_{1}h_{3}\varphi_{c}^{2}+h_{1}^{2}\varphi_{c}^{4} \right]\left[ \mathcal{B}_{1}^{2}+\mathcal{B}_{2}^{2} \right]}$$

$$+\frac{\left[ h_{2}\varphi\sigma_{c} \right]\left[ a_{1}h_{2}\varphi_{c}^{3}-a_{3}h_{2}\varphi_{c}-h_{3}\varphi_{c}^{3}+a_{2}h_{3}\varphi_{c}+h_{1}\varphi_{c}^{5}-a_{2}h_{1}\varphi_{c}^{3} \right]}{\left[ h_{2}^{2}\varphi_{c}^{2}+h_{3}^{2}-2h_{1}h_{3}\varphi_{c}^{2}+h_{1}^{2}\varphi_{c}^{4} \right]\left[ \mathcal{B}_{1}^{2}+\mathcal{B}_{2}^{2} \right]}$$

$$\frac{\begin{aligned} \Longrightarrow3a_{1}h_{1}h_{2}\varphi_{c}^{8}-3a_{3}h_{1}h_{2}\varphi_{c}^{6}-3h_{1}h_{3}\varphi_{c}^{8}+3a_{2}h_{1}h_{3}\varphi_{c}^{6}+3h_{1}^{2}\varphi_{c}^{10}-3a_{2}h_{1}^{2}\varphi_{c}^{8}+3a_{1}h_{2}h_{3}\varphi_{c}^{6} \\ -3a_{1}h_{1}h_{2}\varphi_{c}^{8}-3a_{3}h_{2}h_{3}\varphi_{c}^{4}+3a_{3}h_{1}h_{2}\varphi_{c}^{6}+3h_{2}^{2}\varphi_{c}^{8}-3a_{2}h_{2}^{2}\varphi_{c}^{6}-2a_{1}^{2}h_{1}h_{3}\varphi_{c}^{6}+2a_{1}^{2}h_{1}^{2}\varphi_{c}^{8} \\ +2a_{1}a_{3}h_{1}h_{3}\varphi_{c}^{4}-2a_{1}a_{3}h_{1}^{2}\varphi_{c}^{6}-{2a}_{1}h_{1}h_{2}\varphi_{c}^{8}+{2a}_{1}a_{2}h_{1}h_{2}\sigma_{c}^{6}-2h_{1}^{2}h_{2}^{2}\varphi_{c}^{6}-2h_{1}^{2}h_{3}^{2}\varphi_{c}^{4} \\ +4h_{1}^{3}h_{3}\varphi_{c}^{6}-2h_{1}^{4}\varphi_{c}^{8}+2a_{1}^{2}h_{2}^{2}\varphi_{c}^{6}-2a_{1}a_{3}h_{2}^{2}\varphi_{c}^{4}-2a_{1}h_{2}h_{3}\varphi_{c}^{6}+2a_{1}a_{2}h_{2}h_{3}\varphi_{c}^{4}+2a_{1}h_{1}h_{2}\varphi_{c}^{8} \\ -{2a}_{1}a_{2}h_{1}h_{2}\varphi_{c}^{6}-a_{1}a_{2}h_{1}h_{2}\varphi_{c}^{6}+a_{2}a_{3}h_{1}h_{2}\varphi_{c}^{4}+a_{2}h_{1}h_{3}\varphi_{c}^{6}-a_{2}^{2}h_{1}h_{3}\varphi_{c}^{4}-a_{2}h_{1}^{2}\varphi_{c}^{8}+a_{2}^{2}h_{1}^{2}\varphi_{c}^{6} \\ -3a_{1}h_{2}h_{3}\varphi_{c}^{6}+3a_{3}h_{2}h_{3}\varphi_{c}^{4}+3h_{3}^{2}\varphi_{c}^{6}-3a_{2}h_{3}^{2}\varphi_{c}^{4}-3h_{1}h_{3}\varphi_{c}^{8}+3a_{2}h_{1}h_{3}\varphi_{c}^{6}+2a_{1}^{2}h_{3}^{2}\varphi_{c}^{4} \\ -2a_{1}^{2}h_{1}h_{3}\varphi_{c}^{6}-2a_{1}a_{3}h_{3}^{2}\varphi_{c}^{2}+2a_{1}a_{3}h_{1}h_{3}\varphi_{c}^{4}+2a_{1}h_{2}h_{3}\varphi_{c}^{6}-2a_{1}a_{2}h_{2}h_{3}\varphi_{c}^{4}-a_{1}a_{2}h_{2}h_{3}\varphi_{c}^{4} \\ +a_{1}a_{2}h_{1}h_{2}\varphi_{c}^{6}+a_{2}a_{3}h_{2}h_{3}\varphi_{c}^{2}-a_{2}a_{3}h_{1}h_{2}\varphi_{c}^{4}-a_{2}h_{2}^{2}\varphi_{c}^{6}+a_{2}^{2}h_{2}^{2}\varphi_{c}^{4}+2h_{1}h_{2}^{2}h_{3}\varphi_{c}^{4}+2h_{1}h_{3}^{3}\varphi_{c}^{2} \\ -4h_{1}^{2}h_{3}^{2}\varphi_{c}^{4}+2h_{1}^{3}h_{3}\varphi_{c}^{6}-h_{2}^{4}\varphi_{c}^{4}-h_{2}^{2}h_{3}^{2}\varphi_{c}^{2}+2h_{1}h_{2}^{2}h_{3}\varphi_{c}^{4}-h_{1}^{2}h_{2}^{2}\varphi_{c}^{6}+a_{1}a_{2}h_{2}h_{3}\varphi_{c}^{4} \\ -a_{2}a_{3}h_{2}h_{3}\varphi_{c}^{2}-a_{2}h_{3}^{2}\varphi_{c}^{4}+a_{2}^{2}h_{3}^{2}\varphi_{c}^{2}+a_{2}h_{1}h_{3}\varphi_{c}^{6}-a_{2}^{2}h_{1}h_{3}\varphi_{c}^{4} \end{aligned}}{\left[ h_{2}^{2}\varphi_{c}^{2}+h_{3}^{2}-2h_{1}h_{3}\varphi_{c}^{2}+h_{1}^{2}\varphi_{c}^{4} \right]\left[ \mathcal{B}_{1}^{2}+\mathcal{B}_{2}^{2} \right]}$$

$$\frac{\begin{aligned} \Longrightarrow3h_{1}^{2}\varphi_{c}^{10} \\ +\left[ \begin{aligned} 3a_{1}h_{1}h_{2}-3h_{1}h_{3}-3a_{2}h_{1}^{2}-3a_{1}h_{1}h_{2}+3h_{2}^{2}+2a_{1}^{2}h_{1}^{2} \\ -{2a}_{1}h_{1}h_{2}-2h_{1}^{4}+2a_{1}h_{1}h_{2}-a_{2}h_{1}^{2}-3h_{1}h_{3} \end{aligned} \right]\varphi_{c}^{8} \\ +\left[ \begin{aligned} -3a_{3}h_{1}h_{2}+3a_{2}h_{1}h_{3}+3a_{1}h_{2}h_{3}+3a_{3}h_{1}h_{2}-3a_{2}h_{2}^{2}-2a_{1}^{2}h_{1}h_{3}-2a_{1}a_{3}h_{1}^{2} \\ +{2a}_{1}a_{2}h_{1}h_{2}-2h_{1}^{2}h_{2}^{2}+4h_{1}^{3}h_{3}+2a_{1}^{2}h_{2}^{2}-2a_{1}h_{2}h_{3}-{2a}_{1}a_{2}h_{1}h_{2}-a_{1}a_{2}h_{1}h_{2} \\ +a_{2}h_{1}h_{3}+a_{2}^{2}h_{1}^{2}-3a_{1}h_{2}h_{3}+3h_{3}^{2}+3a_{2}h_{1}h_{3}-2a_{1}^{2}h_{1}h_{3}+2a_{1}h_{2}h_{3} \\ +a_{1}a_{2}h_{1}h_{2}-a_{2}h_{2}^{2}+2h_{1}^{3}h_{3}-h_{1}^{2}h_{2}^{2}+a_{2}h_{1}h_{3} \end{aligned} \right]\varphi_{c}^{6} \\ +\left[ \begin{aligned} -3a_{3}h_{2}h_{3}+2a_{1}a_{3}h_{1}h_{3}-2h_{1}^{2}h_{3}^{2}-2a_{1}a_{3}h_{2}^{2}+2a_{1}a_{2}h_{2}h_{3}+a_{2}a_{3}h_{1}h_{2} \\ -a_{2}^{2}h_{1}h_{3}+3a_{3}h_{2}h_{3}-3a_{2}h_{3}^{2}+2a_{1}^{2}h_{3}^{2}+2a_{1}a_{3}h_{1}h_{3}-2a_{1}a_{2}h_{2}h_{3} \\ -a_{1}a_{2}h_{2}h_{3}-a_{2}a_{3}h_{1}h_{2}+a_{2}^{2}h_{2}^{2}+2h_{1}h_{2}^{2}h_{3}-4h_{1}^{2}h_{3}^{2}-h_{2}^{4}+2h_{1}h_{2}^{2}h_{3} \\ +a_{1}a_{2}h_{2}h_{3}-a_{2}h_{3}^{2}-a_{2}^{2}h_{1}h_{3} \end{aligned} \right]\varphi_{c}^{4} \\ +\left[ -2a_{1}a_{3}h_{3}^{2}+a_{2}a_{3}h_{2}h_{3}+2h_{1}h_{3}^{3}-h_{2}^{2}h_{3}^{2}-a_{2}a_{3}h_{2}h_{3}+a_{2}^{2}h_{3}^{2} \right]\varphi_{c}^{2} \end{aligned}}{\left[ h_{2}^{2}\varphi_{c}^{2}+h_{3}^{2}-2h_{1}h_{3}\varphi_{c}^{2}+h_{1}^{2}\varphi_{c}^{4} \right]\left[ \mathcal{B}_{1}^{2}+\mathcal{B}_{2}^{2} \right]}$$

$$\frac{\begin{aligned} \Longrightarrow3h_{1}^{2}\varphi_{c}^{10}-6h_{1}h_{3}\varphi_{c}^{8}-4a_{2}h_{1}^{2}\varphi_{c}^{8}+2a_{1}^{2}h_{1}^{2}\varphi_{c}^{8}+3h_{2}^{2}\varphi_{c}^{8}-2h_{1}^{4}\varphi_{c}^{8}+4a_{2}h_{1}h_{3}\varphi_{c}^{6} \\ -4a_{2}h_{2}^{2}\varphi_{c}^{6}-4a_{1}^{2}h_{1}h_{3}\varphi_{c}^{6}-2a_{1}a_{3}h_{1}^{2}\varphi_{c}^{6}-3h_{1}^{2}h_{2}^{2}\varphi_{c}^{6}+4h_{1}^{3}h_{3}\varphi_{c}^{6}+2a_{1}^{2}h_{2}^{2}\varphi_{c}^{6}+4a_{2}h_{1}h_{3}\varphi_{c}^{6} \\ +a_{2}^{2}h_{1}^{2}\varphi_{c}^{6}+3h_{3}^{2}\varphi_{c}^{6}+2h_{1}^{3}h_{3}\varphi_{c}^{6}+4a_{1}a_{3}h_{1}h_{3}\varphi_{c}^{4}-6h_{1}^{2}h_{3}^{2}\varphi_{c}^{4}-2a_{1}a_{3}h_{2}^{2}\varphi_{c}^{4}-{2a}_{2}^{2}h_{1}h_{3}\varphi_{c}^{4} \\ 4a_{2}h_{3}^{2}\varphi_{c}^{4}+2a_{1}^{2}h_{3}^{2}\varphi_{c}^{4}+a_{2}^{2}h_{2}^{2}\varphi_{c}^{4}+4h_{1}h_{2}^{2}h_{3}\varphi_{c}^{4}-h_{2}^{4}\sigma_{c}^{4}-2a_{1}a_{3}h_{3}^{2}\varphi_{c}^{2}+2h_{1}h_{3}^{3}\varphi_{c}^{2} \\ +a_{2}^{2}h_{3}^{2}\varphi_{c}^{2}+a_{2}^{2}h_{3}^{2}\varphi_{c}^{2} \end{aligned}}{\left[ h_{2}^{2}\varphi_{c}^{2}+h_{3}^{2}-2h_{1}h_{3}\varphi_{c}^{2}+h_{1}^{2}\varphi_{c}^{4} \right]\left[ \mathcal{B}_{1}^{2}+\mathcal{B}_{2}^{2} \right]}$$

$$\frac{\begin{aligned} \Longrightarrow3h_{2}^{2}\varphi_{c}^{8}+3h_{3}^{2}\varphi_{c}^{6}-6h_{1}h_{3}\varphi_{c}^{8}+3h_{1}^{2}\varphi_{c}^{10}+2a_{1}^{2}h_{2}^{2}\varphi_{c}^{6}+2a_{1}^{2}h_{3}^{2}\varphi_{c}^{4}-4a_{1}^{2}h_{1}h_{3}\varphi_{c}^{6} \\ +2a_{1}^{2}h_{1}^{2}\varphi_{c}^{8}-4a_{2}h_{2}^{2}\varphi_{c}^{6}-4a_{2}h_{3}^{2}\varphi_{c}^{4}+8a_{2}h_{1}h_{3}\varphi_{c}^{6}-4a_{2}h_{1}^{2}\varphi_{c}^{8}-2h_{1}^{2}h_{2}^{2}\varphi_{c}^{6} \\ -2h_{1}^{2}h_{3}^{2}\varphi_{c}^{4}+4h_{1}^{3}h_{3}\varphi_{c}^{6}-2h_{1}^{4}\varphi_{c}^{8}+a_{2}^{2}h_{2}^{2}\varphi_{c}^{4}+a_{2}^{2}h_{3}^{2}\varphi_{c}^{2}-{2a}_{2}^{2}h_{1}h_{3}\varphi_{c}^{4}+a_{2}^{2}h_{1}^{2}\varphi_{c}^{6} \\ -2a_{1}a_{3}h_{2}^{2}\varphi_{c}^{4}-2a_{1}a_{3}h_{3}^{2}\varphi_{c}^{2}+4a_{1}a_{3}h_{1}h_{3}\varphi_{c}^{4}-2a_{1}a_{3}h_{1}^{2}\varphi_{c}^{6}+2h_{1}h_{2}^{2}h_{3}\varphi_{c}^{4} \\ +2h_{1}h_{3}^{3}\varphi_{c}^{2}-4h_{1}^{2}h_{3}^{2}\varphi_{c}^{4}+2h_{1}^{3}h_{3}\varphi_{c}^{6}-h_{2}^{4}\varphi_{c}^{4}-h_{2}^{2}h_{3}^{2}\varphi_{c}^{2}+2h_{1}h_{2}^{2}\varphi_{c}^{4}-h_{1}^{2}h_{2}^{2}\varphi_{c}^{6} \end{aligned}}{\left[ h_{2}^{2}\varphi_{c}^{2}+h_{3}^{2}-2h_{1}h_{3}\varphi_{c}^{2}+h_{1}^{2}\varphi_{c}^{4} \right]\left[ \mathcal{B}_{1}^{2}+\mathcal{B}_{2}^{2} \right]}$$

$$\frac{\begin{aligned} \Longrightarrow3\varphi_{c}^{6}\left[ h_{2}^{2}\varphi_{c}^{2}+h_{3}^{2}-2h_{1}h_{3}\varphi_{c}^{2}+h_{1}^{2}\varphi_{c}^{4} \right]+2a_{1}^{2}\varphi_{c}^{4}\left[ h_{2}^{2}\varphi_{c}^{2}+h_{3}^{2}-2h_{1}h_{3}\varphi_{c}^{2}+h_{1}^{2}\varphi_{c}^{4} \right] \\ -4a_{2}\varphi_{c}^{4}\left[ h_{2}^{2}\varphi_{c}^{2}+h_{3}^{2}-2h_{1}h_{3}\varphi_{c}^{2}+h_{1}^{2}\varphi_{c}^{4} \right]-2h_{1}^{2}\varphi_{c}^{4}\left[ h_{2}^{2}\varphi_{c}^{2}+h_{3}^{2}-2h_{1}h_{3}\varphi_{c}^{2}+h_{1}^{2}\varphi_{c}^{4} \right] \\ {+a}_{2}^{2}\varphi_{c}^{2}\left[ h_{2}^{2}\varphi_{c}^{2}+h_{3}^{2}-2h_{1}h_{3}\varphi_{c}^{2}+h_{1}^{2}\varphi_{c}^{4} \right]-2a_{1}a_{3}\varphi_{c}^{2}\left[ h_{2}^{2}\varphi_{c}^{2}+h_{3}^{2}-2h_{1}h_{3}\varphi_{c}^{2}+h_{1}^{2}\varphi_{c}^{4} \right] \\ +2h_{1}h_{3}\varphi_{c}^{2}\left[ h_{2}^{2}\varphi_{c}^{2}+h_{3}^{2}-2h_{1}h_{3}\varphi_{c}^{2}+h_{1}^{2}\varphi_{c}^{4} \right]-h_{2}^{2}\varphi_{c}^{2}\left[ h_{2}^{2}\varphi_{c}^{2}+h_{3}^{2}-2h_{1}h_{3}\varphi_{c}^{2}+h_{1}^{2}\varphi_{c}^{4} \right] \end{aligned}}{\left[ h_{2}^{2}\varphi_{c}^{2}+h_{3}^{2}-2h_{1}h_{3}\varphi_{c}^{2}+h_{1}^{2}\varphi_{c}^{4} \right]\left[ \mathcal{B}_{1}^{2}+\mathcal{B}_{2}^{2} \right]}$$

$$\frac{\begin{aligned} \Longrightarrow\left[ 3\varphi_{c}^{6}+2a_{1}^{2}\varphi_{c}^{4}-4a_{2}\varphi_{c}^{4}-2h_{1}^{2}\varphi_{c}^{4}{+a}_{2}^{2}\varphi_{c}^{2}-2a_{1}a_{3}\varphi_{c}^{2}+2h_{1}h_{3}\varphi_{c}^{2}-h_{2}^{2}\varphi_{c}^{2} \right] \\ \left[ h_{2}^{2}\varphi_{c}^{2}+h_{3}^{2}-2h_{1}h_{3}\varphi_{c}^{2}+h_{1}^{2}\varphi_{c}^{4} \right] \end{aligned}}{\left[ h_{2}^{2}\varphi_{c}^{2}+h_{3}^{2}-2h_{1}h_{3}\varphi_{c}^{2}+h_{1}^{2}\varphi_{c}^{4} \right]\left[ \mathcal{B}_{1}^{2}+\mathcal{B}_{2}^{2} \right]}$$

$$\frac{\Longrightarrow3\varphi_{c}^{6}+2\left( a_{1}^{2}-2a_{2}-h_{1}^{2} \right)\varphi_{c}^{4}+\left( a_{2}^{2}-2a_{1}a_{3}+2h_{1}h_{3}-h_{2}^{2} \right)\varphi_{c}^{2}}{\mathcal{B}_{1}^{2}+\mathcal{B}_{2}^{2}}$$

Remember that $a_{1}^{2}-2a_{2}-h_{1}^{2}=\mathcal{k}_{1} and a_{2}^{2}-2a_{1}a_{3}+2h_{1}h_{3}-h_{2}^{2}=\mathcal{k}_{2}$, then we have;

$$\frac{d\zeta}{d\tau}|_{\tau{=\tau}_{c}}=\frac{{3\varphi}_{c}^{6}+2\mathcal{k}_{1}\varphi_{c}^{4}+\mathcal{k}_{2}\varphi_{c}^{2}}{\mathcal{B}_{1}^{2}+\mathcal{B}_{2}^{2}}\neq0 (45)$$

Therefore, we conclude that Hopf bifurcation occurs when $\tau$ passes through the critical value, which is assumed to be $\tau_{c}.$
