# Supplementary material for: Fractional-order differential model for knee implant recovery in smart health infrastructures
Source: Sci Rep. 2026 Apr 17;16:17893. doi: 10.1038/s41598-026-48965-7 (PMC13249972; doi:10.1038/s41598-026-48965-7)
Supplement: Supplementary file 2 — Supplementary Material 2 [file 41598_2026_48965_MOESM2_ESM.docx]

Figures S1–S6 illustrate the impact of the fractional order $\mathcal{q}$, inflammation levels, delay, and sensing device usage on knee implant recovery, inflammation, and mechanical function dynamics, as modelled by the fractional-order delay differential equation (7). Figures S1(a–c) show recovery dynamics without sensing devices. Under low inflammation and no delay (Fig. S1a), recovery trajectories rise steadily across all $\mathcal{q}$-values, with higher $\mathcal{q}$corresponding to faster and more complete recovery, highlighting the positive influence of memory effects. Under high inflammation without delay (Fig. S1b), recovery peaks early before declining, with higher $\mathcal{q}$-values maintaining superior performance, demonstrating sensitivity to inflammation and memory. When delay is present under low inflammation (Fig. S1c), recovery is slower but steadily increases, illustrating the dampening effect of delay. Overall, higher $\mathcal{q}$enhances recovery, while delay and inflammation modulate system responsiveness. Figures S2(a–c) present inflammation dynamics without sensing devices. With low inflammation and no delay (Fig. S2a), inflammation declines rapidly, with higher $\mathcal{q}$-values achieving faster suppression. For high inflammation without delay (Fig. S2b), inflammation initially rises before gradually decreasing, with higher $\mathcal{q}$aiding quicker control, while lower $\mathcal{q}$ slows suppression. Under delay and low inflammation (Fig. S2c), decline is gradual, indicating delayed responsiveness. These results emphasize that stronger memory (higher $\mathcal{q}$) improves inflammation regulation, whereas delay and high inflammation challenge system stability. Figures S3(a–c) show mechanical function dynamics without sensing devices. In low inflammation and no delay (Fig. S3a), mechanical function steadily improves, with higher $\mathcal{q}$slightly accelerating recovery. With delay and high inflammation (Fig. S3b), function peaks early then decline, especially at lower $\mathcal{q}$, reflecting reduced implant performance. Under delay with low inflammation (Fig. S3c), function gradually rises and stabilizes across all $\mathcal{q}$, indicating that both memory effects and inflammation levels critically influence long-term mechanical performance. Figures S4(a–c) illustrate recovery dynamics with sensing devices. In low inflammation without delay (Fig. S4a), recovery increases steadily, with higher $\mathcal{q}$accelerating improvement. Under delay and high inflammation (Fig. S4b), recovery rises sharply, particularly for higher $\mathcal{q}$, suggesting sensing devices mitigate inflammatory delays. For delay and low inflammation (Fig. S4c), recovery is linear and consistent, again favoring higher $\mathcal{q}$. These results highlight that sensing devices enhance recovery, especially under delayed or inflammatory conditions, while fractional-order memory further modulates efficiency. Figures S5(a–c) present inflammation dynamics with sensing devices. In low inflammation without delay (Fig. S5a), inflammation peaks around $t\approx500$before declining, with lower $\mathcal{q}$maintaining higher levels, indicating weaker memory-driven suppression. With delay and high inflammation (Fig. S5b), inflammation sharply declines, occasionally reaching negative values, reflecting overcompensation in the inflammatory response, particularly at lower $\mathcal{q}$. Under delay and low inflammation (Fig. S5c), inflammation dips then recover, with higher $\mathcal{q}$achieving faster and more balanced suppression. Overall, sensing devices stabilize inflammation, with higher $\mathcal{q}$improving regulatory response. Figures S6(a–c) depict mechanical function with sensing devices. In low inflammation without delay (Fig. S6a), function rapidly rises and stabilizes, slightly faster for higher $\mathcal{q}$. Under delay and high inflammation (Fig. S6b), function peaks early then decline, more sharply for lower $\mathcal{q}$, showing the negative effects of delay and inflammation. With delay and low inflammation (Fig. S6c), function increases modestly and stabilizes, with higher $\mathcal{q}$performing slightly better. These results suggest that sensing devices support mechanical function, particularly with stronger memory effects, while delay and inflammation challenge long-term stability. Overall, the figures confirm that the fractional order $\mathcal{q}$is critical in modulating recovery, inflammation, and mechanical function. Higher $\mathcal{q}$values enhance memory-driven responsiveness and system resilience, while delays and high inflammation reduce effectiveness unless countered by sensing device feedback.

Figure S1(a – c): Dynamics of the knee implant recovery as in fractional order delay differential equation model (7) with non-usage of sensing devices in the presence and absence of delay with high and low inflammation, $\left( \mathcal{q}=0.976, 0.876, 0.776, 0.676, and 0.576 \right)$.

Figure S2(a – c): Dynamics of the knee implant inflammation as in fractional order delay differential equation model (7) non-usage of sensing devices in the presence and absence of delay with high and low inflammation $\left( \mathcal{q}=0.976, 0.876, 0.776, 0.676, and 0.576 \right)$.

Figure S3(a – c): Dynamics of the knee implant mechanical function as in fractional order delay differential equation model (7) with non-usage of sensing devices in the presence and absence of delay with high and low inflammation $\left( \mathcal{q}=0.976, 0.876, 0.776, 0.676, and 0.576 \right)$.

Figure S4(a – c): Dynamics of the knee implant recovery as in fractional order delay differential equation model (7) with usage of sensing devices in the presence and absence of delay with high and low inflammation, $\left( \mathcal{q}=0.976, 0.876, 0.776, 0.676, and 0.576 \right)$.

Figure S5(a – c): Dynamics of the knee implant inflammation as in the fractional order delay differential equation model (7), usage of sensing devices in the presence and absence of delay with high and low inflammation $\left( \mathcal{q}=0.976, 0.876, 0.776, 0.676, and 0.576 \right)$.

Figure S6(a – c): Dynamics of the knee implant mechanical function as in a fractional order delay differential equation model (7) with usage of sensing devices in the presence and absence of delay with high and low inflammation $\left( \mathcal{q}=0.976, 0.876, 0.776, 0.676, and 0.576 \right)$.
